# 「ばかり」の「限定」と遊離数量詞

**DOI:** 10.12688/f1000research.55582.1

**Published:** 2021-12-14

**Authors:** 大塚貴史 大塚, 白川稜 白川, 橋本修 橋本, 沼田善子 沼田

**Affiliations:** 1 Faculty of Humanities and Social Sciences, University of Tsukuba, Tsukuba, Ibaraki, 305-8571, Japan; 2 Doctoral Program in Literature and Linguistics, Graduate School of Humanities and Social Sciences, University of Tsukuba, Tsukuba, Ibaraki, 305-8571, Japan

**Keywords:** とりたて詞, ばかり, 遊離数量詞, 限定, Toritate focus particle, bakari, exclusivity, floating quantifiers

## Abstract

本稿は，とりたて詞「ばかり」の意味を再考する記述的研究である。従来とりたて詞「ばかり」は限定を表す一方で非該当例を許容する特徴を持つことが指摘されてきた。このため「ばかり」の意味を限定ではないと示唆・主張する研究も見られる。これに対し，本稿では，「ばかり」が遊離数量詞と共起する場合に非該当例を許容しない現象の分析から，「ばかり」の意味を「限定」とすべきであると主張する。本稿では，先行研究を踏まえ，以下のことを明らかにする。「ばかり」の意味解釈のために話者が認知的経験記憶により設定する主観的な集合は，必ずしも現実世界と一致しない場合があるが，遊離数量詞の特徴により，これと「ばかり」が共起する場合は，当該の集合を形成する事態の数量が，現実世界の事態の数量と常に一致する解釈となる。この際，「ばかり」は非該当例を許容しない。従来指摘された非該当例が許容される解釈は，話者が設定する集合が現実世界より狭い範囲で設定され，当該集合の外部に非該当例が存在する場合の解釈と考えるべきである。このことから「ばかり」の意味は非該当例を許容しない「限定」と考えるのが妥当である。

## Abstract (en)

This paper presents a descriptive study that analyzes the semantic meaning of *Toritate* focus particle *bakari*. Previous studies reported that, although *bakari* expresses exclusivity, it is characterized by the fact that it permits non-applicable cases, thereby drawing the conclusion that the meaning of *bakari* is not exclusivity. This paper argues that *bakari* does indeed denote “exclusivity” as *bakari* is supported by the phenomenon that non-applicable cases are unacceptable when *bakari* co-occurs with floating quantifiers. Considering existing research on this subject, the following was observed. Even though the subjective set, as established by the speaker’s past experiences to interpret the meaning of *bakari*, may not be consistent with the real world, the number of events that form the said set match the number of real-world events when *bakari* co-occurs with floating quantifiers due to the characteristics of floating quantifiers. In such cases, *bakari* does not permit non-applicable cases. The interpretation that permits non-applicable cases applies to situations where the set established by the speaker is fixed at a narrower range than the real world, and the non-applicable cases exist outside the set. We thus conclude that *bakari* denotes “exclusivity” that does not permit non-applicable cases.

## 1.　はじめに

とりたて詞「ばかり」は，「だけ」「しか」と同様に限定を表すとされるが，一見するとその位置づけに検討の余地があることを示すような特徴を持つ。それは，非該当例 ^1^を許容するという特徴である。例えば，次の文において，白いシャツ以外（例えば赤いシャツ）も購入している，あるいは三毛猫以外（例えば黒猫）も集まっている場合，「だけ」や「しか」を含む (1ab) (2ab) は成立しないのに対し，「ばかり」を含む (1c) (2c) は成立する。

(1) 【白いシャツを 900 枚，赤いシャツを 100 枚購入した場合】
  a. # 白いシャツ だけを購入した。 ^2^
  b. # 白いシャツ しか購入しなかった。
  c. 白いシャツ ばかりを購入した。
(2) 【三毛猫が 40 匹，黒猫が 10 匹集まった場合】
  a. # 三毛猫 だけが集まった。
  b. # 三毛猫 しか集まらなかった。
  c. 三毛猫 ばかりが集まった。

これは，「だけ」「しか」は非該当例を許容しないのに対し，「ばかり」は非該当例を許容するということを示している。「ばかり」がこうした特徴を持つことは既に指摘されており，それを踏まえて「ばかり」は限定を表すものではないと指摘する研究もある。

しかし，「ばかり」は環境を問わず非該当例を許容するわけではない。例えば，(1c) (2c) に遊離数量詞 ^3^を加えた次の文は，(1) (2) と同様の場合には成立しない。

(3) 【白いシャツを 900 枚，赤いシャツを 100 枚購入した場合】
# 白いシャツ ばかりを 1000 枚購入した。
(4) 【三毛猫が 40 匹，黒猫が 10 匹集まった場合】
# 三毛猫 ばかりが 50 匹 集まった。

(3) (4) は作例であるが、コーパスにおいても「ばかり」に数量詞が後続した文が存在し、これらについての母語話者の内省判断において (3) (4) と同様に非該当例が許容されないことを確認している ^4^。このことは，「ばかり」は遊離数量詞と共起する場合には非該当例を許容しないということを示している ^5^。本稿は，現代語を対象とした記述言語学的研究の一環で，コーパスのデータと母語話者の内省に基づき，この現象について，先行研究の指摘から導き出される遊離数量詞の特徴と関連づけて考察し，その要因を明らかにするものである。さらに，これが「ばかり」の意味について示唆的な現象であることを指摘し，その意味について考察する。分析にあたっては，注 4 ほかで触れたとおり，「現代日本語書き言葉均衡コーパス」(BCCWJ) において本稿で扱う遊離数量詞と共起する「ばかり」に該当する用例，および作例の意味解釈について，日本語母語話者である筆者らの言語直観で判断する。本稿の主張は次の通りである。

(5) a. 遊離数量詞は，事態の数量を表す（数量を事態の数量として表し直す）。
b. 「ばかり」は，遊離数量詞が共起することで，話者による主観的な集合を形成する事態の数量が，現実世界の事態の数量と一致する解釈となるため，結果的に非該当例が存在する可能性を排除する。
(6) とりたて詞「ばかり」の意味は限定であり，非該当例は「ばかり」が問題にする集合 ^6^の外部においてのみその存在が許容され得る。

## 2.　先行研究の指摘と本稿の主眼

「ばかり」が非該当例を許容することは，従来様々な研究において指摘されてきた（ 菊地 1983； 西村 1994； 定延 2001； 澤田 2007； 日本語記述文法研究会編 2009； 佐藤 2017 など）。以下では，その要因についても言及している 定延 (2001) と 佐藤 (2017) を取り上げ，それぞれの指摘を概観した上で本稿の主眼とするところを明確にする。

### 2.1　定延 （2001） の指摘

まず， 定延 (2001) の指摘を概観する。 定延 (2001) は，「探索」という概念を用いて「ばかり」について考察している。「探索」とは「認知領域の拡大行動」（ 定延 2001: 118）であるが， 定延 (2001) は，「ばかり」にはその「探索」が「二重に関わってくる」( 定延 2001: 135) と指摘している。例えば，「ばかり」を含む次の (7) の文の場合，初めに (8a) のような，次に (8b) のような「探索」が行われるとされる。

(7) この人物が食べたのはミカン ばかりだ （ 定延 2001: 129，下線は筆者）
(8) a. 【探索①】問題の人物が食べたモノを探索領域とし，[品種は何か]を探索課題とする探索 ^7^ （ 定延 2001: 129，【 】内は筆者）
b. 【探索②】 探索の集合を探索領域としてそれらが どういう探索なのか，〔筆者略〕1 探索ずつスキャニング探索 （ 定延 2001: 129，下線と【 】内は筆者）

その上で，(7) の文はこの「二重」の「探索」のうち，(8b) の「探索」によって次のような結果が得られたことを表現しているとされる。

(9) 【探索②の結果】すべて［ミカン］という情報を得た 探索だ （ 定延 2001: 129，下線と【 】内は筆者）

定延 (2001) の議論において重要となるのは，「ばかり」を含む文が (8b) の「探索」の結果を表現するという点である。(8a)と (8b) の「探索」は，前者が「世界のありさま」を「探索領域」とするのに対し，後者は「世界探索の集合のありさま」を「探索領域」とするという点で異なるが（ 定延 2001: 134）， 定延 (2001) によれば，後者の場合は非該当例の有無は大きな問題にならないとされる。 定延 (2001)は，次の (10) の例を基に (11) のように述べている。

(10) 先週はうどん ばかり食べた （ 定延 2001: 134，下線は筆者）
(11) 「先週食べたものはうどんがすべてなのか，それともうどんは大部分にすぎず他に何か食べたのか」という問題は，世界のありさまを表現する場合は大きな問題で，仮にうどんが大部分にすぎないにもかかわらず「うどんがすべて」と表現すれば誤りになる。しかし， 世界探索の集合がどのような集合であるかを表現する際には，世界じたいについては，多少印象的・感覚的になっても問題ではなく，「うどんをやたら多く見出す世界探索の集合」であることに変わりはないとしてしまえる ^原注20^。 ( 定延 2001: 134，下線は筆者)

### 2.2　佐藤 （2017） の指摘

これに対し， 佐藤 (2017) は「探索の領域が〔筆者略〕探索という行動の集合である場合に，多少は印象的・感覚的であってもよいという説明に，妥当性はあるのだろうか」（ 佐藤 2017: 7）と疑問を呈し，「ばかり」が非該当例を許容する要因について， 定延 (2001) とは異なる議論を展開している。

佐藤 (2017) は，「認識的際立ち性」という観点から「ばかり」の振る舞いを説明している。 佐藤 (2017) によれば，「認識的際立ち性」とは次のようなものである。

(12) ここ〔筆者注： 佐藤 (2017)〕で言う「認識的際立ち性」とは，当該の主体にとって何らかの意味において容易に捉えられるもの，捉えずにはいられない際立ちをもつものである。 （ 佐藤 2017: 9）

佐藤 (2017) は，集合を問題にする言語形式には，予め確立されている客観的な集合だけでなく，話者の経験に根差して形成された主観的な集合に関与するものがあると述べ（ 佐藤 2017: 8），その一例として「ばかり」を挙げている。また，後者の集合が形成されるに当たっては様々な動機があり得るとしており（ 佐藤 2017: 4-5），特に「ばかり」が関与する集合が形成される動機となるのが「認識的際立ち性」であると指摘している（ 佐藤 2017: 9）。

佐藤 (2017) によれば，「ばかり」が用いられるに当たっては，「認識的際立ち性という動機づけに支えられ，その特徴を有する事態のみを成員とする経験記憶の集合が形成される」（ 佐藤 2017: 9）とされる。例えば， 佐藤 (2017) は次の (13) の文が発話されるに至る過程を (14) のようにまとめている。

(13) あなた，学校に遅刻して ばかりでどうするの （ 佐藤 2017: 3，傍点を下線に改変）
(14) 「ばかり」の集合形成の事例②
  a. 母親が娘の登校時間を気にしながら日常生活を送る。
  b. 週 2 回のペースで娘の学校への遅刻という認識的際立ち性を有する事態を知覚する。
  c. 「娘の遅刻」という認識的際立ち性を有する事態のみから成る経験記憶の集合が形成され，「遅刻してばかり」という認識にいたる。（ 佐藤 2017: 10，下線は筆者）

仮に，週 6 日制の学校に「週 2 回のペース」で遅刻した場合，週 4 回は遅刻していないことになり，(13) の文においてはそれが非該当例となる。しかし，「認識的際立ち性」という特徴を持つもので構成される主観的な集合には「遅刻」のみが含まれる，言い換えれば「非遅刻」は含まれないため ^8^，(13) の文が問題なく成立するとされるのである。

### 2.3　本稿の主眼

以上， 定延 (2001) と 佐藤 (2017) の議論を概観した。いずれにおいても「ばかり」が非該当例を許容する要因について興味深い指摘が見られるが， 佐藤 (2017) も述べているように， 定延 (2001) の指摘には検討の余地がある。これを踏まえ，本稿では「ばかり」が非該当例を許容する要因について， 佐藤 (2017) の考えを採る ^9^。

一方で，「ばかり」と非該当例の関係については，従来考察の対象とされていない問題がある。それは，「ばかり」が非該当例を許容しない環境があるということである。例えば，次の文はいずれも「ばかり」を含むため，先行研究に倣えば非該当例（「赤いシャツ」「黒猫」）が存在していても成立することが予測されるが，(15b) (16b) については成立しない。

(15) 【白いシャツを 900 枚，赤いシャツを 100 枚購入した場合】
  a. 白いシャツ ばかりを購入した。 (=(1a))
  b. # 白いシャツ ばかりを 1000 枚購入した。 (=(3))
(16) 【三毛猫が 40 匹，黒猫が 10 匹集まった場合】
  a. 三毛猫 ばかりが集まった。 (=(2a))
  b. # 三毛猫 ばかりが 50 匹集まった。 (=(4))

(15a) (16a) と (15b) (16b) の相違点は，後者には数量詞が生起しているという点である。これは，一見すると「ばかり」が数量詞と共起する場合は非該当例が許容されないということを示しているように見える。ただし，「ばかり」と数量詞が共起していても，非該当例が許容される場合もある。例えば，次の文ではいずれも「ばかり」と数量詞が共起しているが，非該当例（「男性」）が存在する場合，(17a) は成立しないのに対し，(17b) は成立する。

(17) 【女性を 400 人，男性を 100 人招待した場合】
  a. # 女性 ばかり 500人招待した。
  b. 招待した 500 人は女性 ばかりだ。

つまり，「ばかり」と数量詞が共起していても，(15b) (16b) (17a) は非該当例を許容しないのに対し，(17b)はそれを許容するということになるが，これらは数量詞のタイプが異なる。先行研究では，(15b) (16b)(17a) の数量詞は，(17b) の数量詞に対して遊離数量詞と呼ばれて区別されている。この点を踏まえると，(15) (16) (17) は次のことを示していると言える。

(18) 「ばかり」は非該当例を許容し得るが，遊離数量詞と共起する場合はそれを許容しない。

前述の通り，先行研究では「ばかり」が非該当例を許容することやその要因については指摘されてきたが，「ばかり」がそれを許容しない環境があることについて指摘・考察した研究は管見の限り存在しない。従って，本稿ではこの (18) の現象の解明を主眼とし，その要因を明らかにする（3節）。さらに，この現象を踏まえて「ばかり」の意味についても考察する（4節）。

## 3. 「ばかり」と遊離数量詞

まず，(18) の現象の要因について考察する。以下では，この現象を説明するに当たって重要となる「ばかり」の特徴，及び遊離数量詞の特徴について確認し （3.1 節，3.2 節），それらを踏まえてこの現象の要因を明らかにする（3.3 節）。

### 3.1　事態（の数）から見る非該当例の許容

まず， 佐藤 (2017) の議論の中で特に (18) の現象と密接に関わると考えられる指摘を確認する。前述の通り， 佐藤 (2017) は「ばかり」と「認識的際立ち性」の関わりを指摘しているが（2.2 節），特に「ばかり」が問題にする集合について次のように述べている。

(19) 認識的際立ち性という動機づけに支えられ，その特徴を有する 事態のみを成員とする経験記憶の集合が形成される。 （ 佐藤 2017: 9，下線は筆者）

また， 佐藤 (2017) は「認識的際立ち性」が生じる要因の 1 つとして次の (20a) を挙げ，これについて (20b) のように述べている。

(20) a. 知覚経験される事態の数が 多い。最低でも複数である。 （ 佐藤 2017: 11，下線は筆者）
b. 一度の失敗しか経験されていない場合，「失敗ばかり」とは言えないだろう。したがって，この要因は 「ばかり」が使われるための必要条件である ^原注6^。 （ 佐藤 2017: 11，下線は筆者）

このように， 佐藤 (2017) は「ばかり」が問題にする集合に含まれるのは「事態」であり，その数が「多い」ことが「ばかり」が用いられる要件であると指摘している。このことは次のようにまとめられる。

(21) a. 「ばかり」は 事態を問題にする。 ^10^
b. 「ばかり」は事態の数が 多いと認識されれば用いられ得る。

次に，この (21) に注目しつつ，「ばかり」が非該当例を許容する背景について改めて検討する。前述の通り，次の (22) の文は (23) の状況において問題なく成立する。

(22) 白いシャツ ばかりを購入した。
(23) 白いシャツを 900 枚，赤いシャツを 100 枚，計 1000 枚のシャツを購入した。

このとき，「ばかり」が事態を問題にするということ ((21a)) を踏まえると，(22) の文が成立するに当たり，(23) の状況は次のように捉え直されていると考えられる。

(23’) 「白いシャツを購入する」という事態が 900，「赤いシャツを購入する」という事態が 100， 計1000 の「購入する」という事態が生じた。

つまり，(22) の文が成立するということは，事態の総数は 1000 であるものの，「ばかり」はそのうちの 900 の事態 のみを問題にすることが可能ということになる。このとき，(22) では「白いシャツを購入する」という事態の数が多いということは間接的に表現され得るが ^11^，その具体的な数(総数に一致する数なのか，あるいはそれに近い数なのか)には関与していない。つまり，「ばかり」は次のような特徴を持つのであり，これが背景となって非該当例が許容されることになると言える。

(24) 「ばかり」は，（事態の総数が文脈上示されている場合でも）問題にする事態の具体的な数には関与しない。

### 3.2　遊離数量詞と事態の数量

次に，遊離数量詞に関する先行研究の指摘を見る。 矢澤 (1985) は，本稿での遊離数量詞に当たる「NCQ型」の数量詞 ^12^について，「何らかの形で動詞の表す動作・作用に関連した数量を表しているのではないか」（ 矢澤 1985: 104）と述べ ^13^，「NCQ型」の数量詞とそれ以外の数量詞の相違点について次のように指摘している。

(25) NCQ型の数量詞〔筆者注:本稿での遊離数量詞〕は，述部に直接関わり，その述部の表す動作・作用の上で先行名詞句と間接的な意味的関係を結ぶのに対し，NCQ型以外の型の数量詞は，先行名詞句に直接関わり，先行名詞句が述部と関わることによって，数量詞と述部との間接的な関係ができると考えるのである。 （ 矢澤 1985: 105-106，下線は筆者）

この指摘は，遊離数量詞が事態と密接に関わることを示している。具体的には，遊離数量詞は次のような特徴を持つと言える。

(26) 遊離数量詞は，事態の数量を表す（数量を事態の数量として表し直す）。 (=(5a))

### 3.3　遊離数量詞共起下において非該当例が許容されない要因

以上の点を踏まえ，「ばかり」が遊離数量詞と共起する場合に例外を許容しなくなる現象の要因について検討する。次の例を見られたい。

(27) 白いシャツ ばかりを 1000 枚購入した。 (=(3))

前述の通り，「ばかり」はその集合の数量には関与しないが ((24))，遊離数量詞は明示的にその事態の数を表す。(27) の文で言えば，「1000 枚」という遊離数量詞が生起することで，次のようなことが表される。

(28) 問題になる「白いシャツを購入する」という事態の数は 1000 であった。

これにより，「ばかり」が問題にする「白いシャツを購入する」という事態の数が 1000 にいわば固定され，結果的にその中に他の事態（「赤いシャツを購入する」など）が存在する余地がなくなるのである。つまり，「ばかり」が遊離数量詞と共起する場合に非該当例を許容しない要因は次のようにまとめられる。

(29) 「ばかり」は，遊離数量詞が共起することで，話者による主観的な集合を形成する事態の数量 ^14^が，現実世界の事態の数量と一致する解釈となるため，結果的に非該当例が存在する可能性を排除する。 (=(5))

## 4. 「ばかり」の意味について

次に，「ばかり」の意味について考察する。以下では，まず，「ばかり」が非該当例を許容する現象に触れる先行研究のうち，「ばかり」の意味にも言及するものの指摘を概観し，併せてその問題点を明らかにする （4.1 節）。その上で，「ばかり」の意味はあくまで限定と捉えるべきであると主張する （4.2 節）。

### 4.1　先行研究の指摘と問題の所在

とりたて詞「ばかり」の意味については既に様々な先行研究において考察されており，多くの場合，「ばかり」は限定を表すとされる（ 丹羽 1992； 益岡・田窪 1992； 中西 1995； 沼田 2009 など）。しかし，特に「ばかり」が非該当例を許容する現象に触れる先行研究においては必ずしもそうではない。以下では，「ばかり」が非該当例を許容する現象に触れつつ「ばかり」の意味についても言及している 日本語記述文法研究会編 (2009) と 澤田 (2007) を取り上げてその指摘を概観し，併せてその問題点を明らかにする。

4.1.1 日本語記述文法研究会編 (2009) の指摘とその問題点

まず， 日本語記述文法研究会編 (2009) は次のように述べ，「ばかり」は限定を表すと主張している。

(30) 「ばかり」は， とりたてた要素が唯一のものであることを示し，ほかのものを排除するという 限定の意味を表す。（ 日本語記述文法研究会編 2009: 61，下線は筆者）

また， 日本語記述文法研究会編 (2009) は次の (31) の文について (32) のように述べ，「ばかり」が非該当例を許容することに触れている。

(31) 佐藤さんは来客にコーヒー ばかり出した。 （ 日本語記述文法研究会編 2009: 62）
(32) コーヒー以外のものも出した可能性は完全には否定されない。 （ 日本語記述文法研究会編 2009: 62）

「ばかり」が限定を表すことと非該当例を許容することには一見すると理論的矛盾がある。しかし， 日本語記述文法研究会編 (2009) によれば，「ばかり」が表す限定には次のような2つの下位分類があり，非該当例が許容される (31) の文では，このうち (33b) のような「限定の仕方」が採られているとされる。

(33) a. とりたてた要素が唯一のものであることを示し，ほかのものを排除するという限定の仕方 （ 日本語記述文法研究会編 2009: 62）
b. とりたてた要素が関わる事態が 何度も繰り返されることや，とりたてた要素が重なって 多数にのぼることを表すという限定の仕方 （ 日本語記述文法研究会編 2009: 62，下線は筆者）

日本語記述文法研究会編 (2009) の指摘は，非該当例の許容という現象について限定という意味の下で説明しようと試みている点で注目に値する。しかし，その説明には不十分な点がある。確かに，(31) の文は「コーヒーを出す」という事態が複数回生じていなければ成立せず，その点で (33b) において述べられているように「何度も繰り返されること」「多数にのぼること」を表していると言える。しかし，その (33b) を (30) の下位分類としていることには問題がある。具体的に言えば，「何度も繰り返されること」「多数にのぼること」((33b))と「唯一のものである」「ほかのものを排除する」((30)) ということには隔たりがある。それにもかかわらず， 日本語記述文法研究会編 (2009) ではその点について特段の言及がなされていないのである。この点に鑑みれば， 日本語記述文法研究会編 (2009) の説明は十分とは言えない ^15^。

4.1.2 澤田 (2007) の指摘とその問題点

これに対し， 澤田 (2007) は「ばかり」の（主たる）意味は限定ではないと主張している。 澤田 (2007) は， 菊地 (1983) が挙げる次の(34)の文について (35) のように述べている。

(34) この一週間そば バカリ食べたよ。 （ 菊地 1983: 58，下線は筆者）
(35) 「ばかり」を使用する第一の目的は，「毎日そばを食べた」とカテゴリーを限定するというより， 話し手が「この一週間を思い起こせば，よくそばを食べた，それは通常の一週間より多すぎた」ということを伝える方が重要であり，その 二次的な効果として明示された要素に対比される要素(明示された要素以外にその現象を成り立たせる可能性のある要素)がその観察された中に少なかった。または，なかったと伝えることになる。 派生的に，限定的解釈がでてくるのである。 （ 澤田 2007: 118-119，下線は筆者）

このように， 澤田 (2007) は，「ばかり」は「通常より多い」ということを表すのであり，限定(的解釈)はそこから「派生」する「二次的な効果」であると捉えている。つまり，限定は「ばかり」の意味ではなく，言わば語用論的効果であるとしているのである。

澤田 (2007) の指摘において注目されるのは，「ばかり」が問題にする集合と非該当例の関係である。(35) では，「ばかり」は場合によっては「明示された要素に対比される要素」が「観察された中に少なかった」ということを伝え得るとされている。これは，「ばかり」が問題にする集合に「明示された要素に対比される要素」が含まれていても構わないということを意味する。 澤田 (2007) の言う「明示された要素に対比される要素」が本稿での非該当例に当たると推察されることを踏まえると， 澤田 (2007) は次のことを示唆していると言える。

(36) 「ばかり」は，問題にする集合に非該当例が含まれていても用いられ得る。 ^16^

しかし，「ばかり」と遊離数量詞が共起した場合の現象を踏まえれば，この (36) は否定せざるを得ない。本稿では，3 節において，非該当例を許容し得る「ばかり」が遊離数量詞と共起した場合にそれを許容しなくなるという現象について考察した。その要因を検討する過程で，遊離数量詞が共起することで，話者による主観的な集合を形成する事態の数量が，現実世界の事態の数量と一致する解釈となるということを指摘したが ((29))，これは次のことを意味する。

(37) 遊離数量詞の共起によって，「ばかり」が問題にする集合（に含まれる事態の数）が遊離数量詞によって示される現実世界の集合と同一の集合に固定される。

仮に， 澤田 (2007) が示唆するように，「ばかり」は問題にする集合に非該当例が含まれていても用いられ得るとすれば，遊離数量詞によって「ばかり」が問題にする集合（に含まれる事態の数）が固定された次の文も，場合によっては「1000」の「購入する」という事態の中に非該当例（「赤いシャツを購入する」）が含まれていても成立するということになるが，次の文がそうした状況下では成立しないことは前述の通りである。

(38) 【白いシャツを 900 枚，赤いシャツを 100 枚購入した場合】
# 白いシャツ ばかりを 1000 枚購入した。 (=(3))

### 4.2 「ばかり」の意味

以上，「ばかり」が非該当例を許容する現象に触れる先行研究における「ばかり」の意味に関する指摘を確認したが，いずれにおいても問題があると言える。これに対し，本稿では「ばかり」の意味について次のように主張する。

(39) とりたて詞「ばかり」の意味は限定であり，非該当例は「ばかり」が問題にする主観的な集合の外部においてのみその存在が許容され得る。 (=(6))

まず，「ばかり」は限定，即ちとりたてた要素（事態）が唯一存在し，他のものを排除する ^17^ということを表すと主張する。前述の通り，「ばかり」の意味が限定でないとすると，遊離数量詞と共起した場合に非該当例が許容されない現象を説明することができないためである。

ただし，その限定は「認識的際立ち性」などに起因して形成される主観的な集合の内部に対してのものである。従って，現実世界において客観的には非該当例が存在していても，それが（「認識的際立ち性」を持たないが故に）「ばかり」が問題にする集合に含まれなければ，「ばかり」は用いられ得るのである。

## 5.　おわりに

本稿では，「ばかり」が遊離数量詞と共起する場合に非該当例が許容されない現象を取り上げ，その現象が，「ばかり」が問題にする集合に含まれる事態の数量が遊離数量詞によって現実世界の事態の数量と一致する解釈になることに起因することを明らかにした。また，これを通して，とりたて詞「ばかり」の意味は限定であると主張した。この主張は多くの先行研究に見られるものであるが，「ばかり」が非該当例を許容することを認めた上でそのように主張する研究はほとんど存在せず ^18^，その点で，「ばかり」は限定を表すと考えざるを得ない現象にも触れつつその主張を明示したことに意義があると考える。

ところで，本稿では， 佐藤 (2017) の指摘を踏まえ，「ばかり」が問題にするのは現実世界を反映する予め確立された客観的な集合ではなく，自己の経験に根差して形成される主観的な集合であると捉えることにより，限定という意味の下で非該当例の許容という現象が説明されると論じた。これは，「ばかり」の意味記述においては，その意味の対象となる集合(以下，対象集合)が重要となることを示しているが，この対象集合という視点の有用性は，「ばかり」の意味記述に限られるものではないと考える。まず挙げられるのは，他のとりたて詞の意味記述に当たっての有用性である。管見の限り，従来のとりたて詞研究で　は，とりたて詞各語について対象集合が詳細に議論されることや，それぞれの対象集合の設定のされ方の異同を本格的に取り上げた考察はほとんど行われていない。他のとりたて詞についても対象集合に関する考察を深めることで，個別のとりたて詞の意味やとりたて詞全体の意味体系の記述の精緻化が可能となろう。また，とりたて詞に留まらず，非該当例を許容しないとされる諸形式の意味記述に当たってもこの視点が有用であると考えられる。　例えば，全称量化詞などと呼ばれる「全部」「みんな」，さらに「常に」「いつも」などは，基本的には非該当例を許容しないとされるが，「みんな」や「いつも」など一部の形式については非該当例を許容し得る。このこと自体は既に 佐藤 (2017) で指摘されており，意味的な観点からその要因を明らかにしようとする研究も存在する（ 大塚 2020， 2021）。しかし，対象集合に注目して再検討することで，先行研究において未だ解明されていない点について説明を与えることが可能になると考える。これらについては稿を改めて論じることとする。

## データ可用性

本論文の研究結果の基礎となるデータは，すべて本論文中に示されており，追加のソースデータは必要とされていない。例外として，注4で示したデータ絞り込みの結果，判断を加えた42例の提示は，国立国語研究所による現代日本語書き言葉均衡コーパス(BCCWJ)「中納言」より入手できる。同コーパス利用には登録が必要だが，他の研究者も著者と同じようにデータにアクセスできる。登録方法については https://chunagon.ninjal.ac.jp/auth/login?service=https%3A%2F%2Fchunagon.ninjal.ac.jp%2Fj_spring_cas_security_check を参照されたい。

## Footnotes

^1^
本稿では，「ばかり」がとりたてる要素に該当しない例（いわゆる「例外」）のことを， 佐藤 (2017) に倣って「非該当例」と呼称する。なお，2.1 節で触れる 定延 (2001) はこれを「夾雑物」と呼称しているが，煩雑化を避けるため，本稿では「非該当例」という用語で統一する。

^2^
先行研究から引用した例文などの末尾にはその出典を記す。一方，出典のないものは筆者によるものであるが，筆者の作例には「#」を付すことがある。これは，当該の文が文法的ではあるものの，指定の文脈では不自然ということを示す記号である。また，引用した例文には「?」「??」を付すことがあるが，これは引用元の文献に倣ったものであり，いずれも当該の文が（やや）不自然であることを示す記号である。

^3^
先行研究では，数量詞の捉え方について幾つかの立場があり，遊離数量詞と呼称すべき範囲，あるいは名称そのものについても議論がある（詳細は 矢澤 (1988) や 加藤 (1997) などを参照されたい）。しかし，本稿ではその点には立ち入らず，副詞位置に生起する数量詞を便宜的に遊離数量詞と呼称する。

^4^
コーパスは 現代日本語書き言葉均衡コーパス (BCCWJ) を使用した。該当文抽出，内省判断の手順は以下の通りである。 ・BCCWJをアプリケーション「中納言」で使用 ・検索・抽出の手順は， 短単位検索 キー：ばかり 後方共起:キーから1語，品詞の小分類が名詞・数詞 →ヒット数 45 例 →上記 45 例を目視で確認，バグ 3 例を除外 →残った 42 例について母語話者により内省判断抽出された BCCWJ 内の文例を 1 例示す。 (i) 今日は、映画の予告編ばかり二十四本見てきました。（サンプルID:OY15_13680、yahoo 知恵袋））

^5^
(3) (4) について，非該当例が認められる場合でも成立すると判断する話者の存在も完全には否定できない。ただし，本稿においてこれらが当該の文脈で成立しないと主張するのは意味論のレベルであるのに対し，成立するという判断は語用論のレベルでなされるものであると考える。語用論の1つのモデルである「関連性理論 (Relevance Theory)」を提唱する Sperber and Wilson (1995) は，「思考の最適な解釈的表現は，聞き手にその思考について処理するに値するだけの関連性がある情報を与え，できるだけ処理労力が少なくてすむようにしなくてはなら」( Sperber and Wilson 1995: 284)ず，「厳密に言えば偽とわかっている」( Sperber and Wilson 1995: 284) 内容でも成立する場合があるとしている。非該当例が認められる場合でも (3) (4) が成立するという判断があり得るとすれば，それはこうした語用論のレベルでの判断であり，本稿が目的とする意味論のレベルの議論とは区別されるべきものである。

^6^
「『ばかり』が問題にする集合」とは， 沼田 (2009) 等で述べる，自者とそれに対する同類の他者が構成する集合である。詳しくは 沼田 (2009: 43-56) を参照されたい。

^7^
「探索領域」は「探索が及ぶ領域」（ 定延 2001: 118）を，「探索課題」は「探索者が探索を通して解決しようとする課題」（ 定延 2001: 119）を意味する。

^8^

佐藤 (2017) は，「認識的際立ち性という性質をよりもちやすくする要因」（ 佐藤 2017: 11） の1つとして次のことを挙げている。

(i) 事態が信念に照らし合わせて 有標的である。 （ 佐藤 2017: 11，下線は筆者）
  この指摘は，次のような文の容認度の差が踏まえられている。
(ii) 太郎は 授業をさぼって ばかりだ。 （ 佐藤 2017: 12，下線は筆者）
(iii) ?? 太郎は 授業に出席して ばかりだ。 （ 佐藤 2017: 12，下線は筆者）

佐藤 (2017) は，「常識的な信念を有するものにとって，『授業をさぼる』〔筆者略〕といった行為はあるまじきものであり，有標性の高いものといえよう」（ 佐藤2017: 12）と述べている。そのために「認識的際立ち性」が生じやすく，(ii) は自然な文となる。一方，(iii) が不自然なのは，「授業に出席する」という事態は「有標性」が低く，「認識的際立ち性」を持ちにくいためであると推察される。「非遅刻」という事態が「ばかり」が問題にする集合に含まれないのも，この事態が「授業に出席する」という事態と同様に「有標性」が低いためであると考えられる。

^9^
なお， 佐藤 (2017) は「本稿〔筆者注： 佐藤 (2017)〕が論じた集合形成の議論における知覚経験という観点は， 定延 (2001) の言うところの『探索』というわれわれの心身の行動を前提とするものであり，その意味で本研究は 定延 (2001) の議論の延長線上に位置づけられる」（ 佐藤 2017: 13）と述べており， 定延 (2001) が提唱する「探索」という行動そのものに異議を唱えているわけではない。これについては本稿も同様である。

^10^
「ばかり」が事態を問題にするということは， 佐藤 (2017) 以前にも示唆・指摘されている。例えば， 森田 (1980) は次の (iv) のように， 菊地 (1983) は (v) のように述べ，「ばかり」と事態の関わりについて言及している。

(iv) 「ばかり」は，“ある同一同類の主体がある範囲 *で行*う”とか，“同一同類の事柄をある範囲内 *で行*う”とか，“同一同類の対象に対して *行われる*”とか，また，“ある同一の事物がある範囲の程度内で *存在する*”とか，あるいは“ある事態に *対応する*のがいつも同じ人物である”とか，いずれの場合も 動詞的叙述(傍点部分(筆者注: 本稿中斜体))を前提としている。 （ 森田 1980: 402，下線は筆者）
(v) バカリは，<同類として括れる 事態が数多くみとめられる>時に使われる。 （ 菊地 1983: 57，下線は筆者）

なお， 定延 (2001) は 菊地 (1983) による(v)の指摘に触れた上で，「『ばかり』の探索領域が事物の集合ではなく，事物を探索領域とする探索の集合であると考える点で，本稿〔筆者注： 定延 (2001)〕は菊地〔筆者注： 菊地 (1983)〕と同じ立場に立つ」（ 定延 2001: 130）と述べている。その点では， 定延 (2001)も「ばかり」は事態に関わると捉えていると言える。

^11^
事態の数が多いということは「ばかり」が用いられる動機となり得るというだけで，「ばかり」が直接的に表現しようとする内容ではない。ただし，それに起因して「ばかり」が用いられることがある ((21b)) 以上，間接的には「ばかり」は事態の数が多いということを表し得ると言える。

^12^

奥津 (1983) 以降の数量詞研究では，しばしば「NCQ型」「NQC型」「NノQC型」「QノNC型」といった名称が用いられる。これらの名称は，数量詞をその現れ方によって分類した際に用いられるものであり，Nが名詞を，Cが格助詞を，Qが数量詞を指している。

^13^
この 矢澤 (1985) の指摘は，「NCQ型の数量詞は，述部が動詞句以外のときには，現れにくいという構文上の制約がある」（ 矢澤 1985: 103）ことに基づいている。 矢澤 (1985) は，述部が動詞句以外である次の文において，「NCQ型」の数量詞を含む (vi) (vii) (viii) とそれ以外の数量詞を含む (ix) (x) (xi) では，前者の方が容認度が低いことを示している。

(vi) ? ココニイル女性ハ 五人　高校生ノ先生ダ （ 矢澤 1985: 103）
(vii) ? アノ会社ノ受付嬢ハ 三人　美シイ （ 矢澤 1985: 103）
(viii) ? アノ台ノ上ニ並ンダ牛乳ハ 五本　古イ （ 矢澤 1985: 103）
(ix) ココニイル女性 五人ハ　高校生ノ先生ダ       （NQC型） （ 矢澤 1985: 103）
(x) アノ会社ノ受付嬢ノ（中ノ） 三人ハ　美シイ       （NノQC型） （ 矢澤 1985: 104）
(xi) アノ台ノ上ニ並ンダ 五本ノ牛乳ハ　古イ        （QノNC型） （ 矢澤1985: 104）

^14^
これまでの議論では事態の「数」が問題となる現象，例をとりあげてきたが，「実験のために，残留塩素濃度が基準値を超える水ばかりを3000cc 集める。」のように「量」が問題となる例もある。これらも含めて扱うため，ここでは事態の「数量」とする。

^15^
なお，2つ提示されている「ばかり」の「限定の仕方」の 1 つである (33a) の説明は，その上位に当たる限定の意味に関する (30) の説明と完全に一致しているが，これはそもそも下位分類の設定として適切とは言い難い。この点も， 日本語記述文法研究会編 (2009) の捉え方に検討の余地があることを示唆している。

^16^

定延 (2001)も， 澤田 (2007) と同じく(36) を示唆しているように読める。 定延 (2001) は，「ばかり」は限定を表すという立場を採っており（ 定延 2001: 113），その点で 澤田 (2007) とは異なる。一方で，「ばかり」が問題にする「世界探索の集合」は印象的・感覚的になっても問題ではなく，多少の非該当例の存在は「ばかり」の使用に影響しないとも指摘している（ 定延 2001: 134）。この点については 澤田 (2007)との類似性が認められると言える。

^17^
この限定についての説明は，「ばかり」に関する先行研究での説明を踏襲したものである。例えば 丹羽 (1992) は，「限定とは，当該事態が唯一成立して他の事態は排除されるということ」（ 丹羽 1992: 109）と述べている。また， 沼田 (2009) は「とりたて詞がとりたてる文中の要素」（ 沼田 2009: 37）を「自者」，それに「端的に対比される『自者』以外の要素」（ 沼田 2009: 37） を「他者」と呼び，「自者」が肯定され，かつ「他者」が否定されることを限定と呼んでいる（ 沼田 2009: 196）。

^18^
そうした研究に当たるものは，管見の限り 日本語記述文法研究会編 (2009) のみである。しかし，その分析に問題があることは 4.1.1 節で述べた通りである。
