# 「ばかり」の「限定」と遊離数量詞

**DOI:** 10.12688/f1000research.55582.2

**Published:** 2022-07-04

**Authors:** 大塚貴史 大塚, 白川稜 白川, 橋本修 橋本, 沼田善子 沼田

**Affiliations:** 1 Faculty of Humanities and Social Sciences, University of Tsukuba, Tsukuba, Ibaraki, 305-8571, Japan; 2 Department of Japanese Language, Faculty of Foreign Languages, Daito Bunka University, Itabashi-ku, Tokyo, 175-8571, Japan; 3 Doctoral Program in Literature and Linguistics, Graduate School of Humanities and Social Sciences, University of Tsukuba, Tsukuba, Ibaraki, 305-8571, Japan; 4 Faculty of Human and Cultural Sciences, Aikoku Gakuen University, Yotsukaido, Chiba, 284-0005, Japan

**Keywords:** とりたて詞, ばかり, 遊離数量詞, 限定, toritate focus particle, bakari, exclusivity, floating quantifiers

## Abstract

本稿は，とりたて詞「ばかり」の意味を再考する記述的研究である。従来とりたて詞「ばかり」は限定を表す一方で非該当例を許容する特徴を持つことが指摘されてきた。このため「ばかり」の意味を限定ではないと示唆・主張する研究も見られる。これに対し，本稿では，「ばかり」が遊離数量詞と共起する場合に非該当例を許容しない現象の分析から，「ばかり」の意味を「限定」とすべきであると主張する。本稿では，先行研究を踏まえ，以下のことを明らかにする。「ばかり」の意味解釈のために話者が認知的経験記憶により設定する主観的な集合は，必ずしも現実世界と一致しない場合があるが，遊離数量詞の特徴により，これと「ばかり」が共起する場合は，当該の集合を形成する事態の数量が，現実世界の事態の数量と常に一致する解釈となる。この際，「ばかり」は非該当例を許容しない。従来指摘された非該当例が許容される解釈は，話者が設定する集合が現実世界より狭い範囲で設定され，当該集合の外部に非該当例が存在する場合の解釈と考えるべきである。このことから「ばかり」の意味は非該当例を許容しない「限定」と考えるのが妥当である。

## Abstract (en)

This paper presents a descriptive study that analyzes the semantic meaning of *toritate *focus particle *bakari*. It has been pointed out that while *bakari *expresses *exclusivity*, it seemingly possesses the characteristic of allowing non-applicable cases. This phenomenon may be examined when one argues against the position that *bakari* expresses *exclusivity*. Contrary to this view, the current study shows that when *bakari* co-occurs with floating quantifiers, non-applicable cases cannot be included in the quantity of the item indicated by the quantifier in question, thereby arguing that the meaning of *bakari* should be taken as *exclusivity*. Based on the previous studies, we clarify the following two points. First, when *bakari *is used, apart from a pre-established objective set reflecting the world, a subjective set rooted in the speaker's experience is established, and when a floating quantifier co-occurs with *bakari*, the quantifier in question expresses the quantity of the subjective set. Second, *toritate *focus particle *bakari *represents *exclusivity,* and the non-applicable cases can exist only outside of the set that *bakari* is concerned with.

## 1.　はじめに

とりたて詞「ばかり」は，「だけ」「しか」と同様に，概ね次のように規定される「限定」を表すとされる。

(1) ある集合の内部において，とりたて詞がとりたてる要素が存在し，それ以外の要素が存在しない ^1^。
  一方で，その位置づけに検討の余地があることを示すような現象が観察される。例えば，次の (2) (3) のように，「白いシャツ」以外も購入した，あるいは「三毛猫」以外も集まったという場合，「だけ」「しか」を含む (2ab) (3ab) は成立しないのに対し，「ばかり」を含む (2c) (3c) は成立し得る。
(2) 【白いシャツを 900 枚，赤いシャツを 100 枚購入した場合】
  a. # 白いシャツ だけを購入した ^2^。
  b. # 白いシャツ しか購入しなかった。
  c. 白いシャツ ばかりを購入した。
(3) 【三毛猫が 40 匹，黒猫が 10 匹集まった場合】
  a. # 三毛猫 だけが集まった。
  b. # 三毛猫 しか集まらなかった。
  c. 三毛猫 ばかりが集まった。

この現象は，「ばかり」はとりたてた要素に該当しない要素（以下，非該当例 ^3^）の存在を許容するということ，さらに「ばかり」は「限定」を表すものではないということを示すかのように見える。

しかし，そうとは言い切れないことを示唆する現象も観察される。例えば， (2c) (3c) に遊離数量詞 ^4^を加えた次の文は， (2) (3) と同様の場合には成立しない。

(4) 【白いシャツを 900 枚，赤いシャツを 100 枚購入した場合】
  # 白いシャツ ばかりを 1000 枚購入した。
(5) 【三毛猫が 40 匹，黒猫が 10 匹集まった場合】
  # 三毛猫 ばかりが 50 匹集まった。

これらの文が成立するためには「1000 枚」のすべてが「白いシャツ」，あるいは「50 匹」のすべてが「三毛猫」でなければならず，「1000 枚」や「50 匹」の中に非該当例（「赤いシャツ」や「黒猫」など）の存在が許容される余地はない ^5^ ^,^ ^6^。

本稿は，現代語を対象とした記述言語学的研究の一環で，コーパスのデータと日本語母語話者の内省に基づき，この現象について先行研究の指摘から導き出される遊離数量詞の特徴と関連づけて考察し，その要因を明らかにするものである。さらに，これが「ばかり」の意味について示唆的な現象であることを指摘し，その意味について考察する。分析に当たっては，『現代日本語書き言葉均衡コーパス』 (BCCWJ) より抽出した用例 ^7^，及び作例の意味解釈について，日本語母語話者である筆者らの言語直観で判断する。本稿の主張は次の通りである。

(6) a. 遊離数量詞は事態の数量を表す （数量を事態の数量として表し直す）。
b. 「ばかり」が遊離数量詞と共起する場合，当該の数量詞は客観的な集合ではなく，「ばかり」が設定する主観的な集合に含まれる事態の数量を表す。
(7) とりたて詞「ばかり」は「限定」を表し，非該当例は「ばかり」が設定する集合 ^8^の外部においてのみその存在が許容され得る。

## 2.　先行研究の指摘と本稿の主眼

「ばかり」と非該当例の関係については，従来様々な研究において指摘されてきた（ 菊地 1983; 西村 1994; 定延 2001; 澤田 2007; 日本語記述文法研究会 2009; 佐藤 2017 など）。以下では，「ばかり」が非該当例を許容する（ように見える）要因についても言及している 定延 (2001) と 佐藤 (2017) を取り上げ，それぞれの指摘を概観した上で本稿の主眼とするところを明確にする。

### 2.1 定延 (2001) の指摘

まず， 定延 (2001) の指摘を概観する。 定延 (2001) は，「探索」という概念を用いて「ばかり」について考察している。「探索」とは「認知領域の拡大行動」（ 定延 2001: 118）であるが， 定延 (2001) は，「ばかり」にはその「探索」が「二重に関わってくる」（ 定延 2001: 135）と指摘している。例えば，「ばかり」を含む次の (8) の文の場合，初めに (9a) のような，次に (9b) のような「探索」が行われるとされる。

(8) この人物が食べたのはミカン ばかりだ（ 定延 2001: 129，下線は筆者）
(9) a. 【探索①】問題の人物が食べたモノを探索領域とし，[品種は何か]を探索課題とする探索 ^9^ （ 定延 2001: 129，【 】内は筆者）
      b. 【探索②】 探索の集合を探索領域としてそれらが どういう探索なのか，〔筆者略〕 1 探索ずつスキャニング探索 （ 定延 2001: 129，下線と【 】内は筆者）

その上で，(8) の文はこの「二重」の「探索」のうち，(9b) の「探索」によって次のような結果が得られたことを表現しているとされる。

(10) 【探索②の結果】すべて [ミカン]という情報を得た 探索だ （ 定延 2001: 129，下線と【 】内は筆者）

定延 (2001) の議論において重要となるのは，「ばかり」を含む文が (9b) の「探索」の結果を表現するという点である。(9a) と (9b) の「探索」は，前者が「世界のありさま」を「探索領域」とするのに対し，後者は「世界探索の集合のありさま」を「探索領域」とするという点で異なるが（ 定延 2001: 134）， 定延 (2001) によれば，後者の場合は非該当例の有無は大きな問題にならないとされる。 定延 (2001) は，次の (11) の例を基に (12) のように述べている。

(11) 先週はうどん ばかり食べた（ 定延 2001: 134，下線は筆者）
(12) 「先週食べたものはうどんがすべてなのか，それともうどんは大部分にすぎず他に何か食べたのか」という問題は，世界のありさまを表現する場合は大きな問題で，仮にうどんが大部分にすぎないにもかかわらず「うどんがすべて」と表現すれば誤りになる。しかし， 世界探索の集合がどのような集合であるかを表現する際には，世界じたいについては，多少印象的・感覚的になっても問題ではなく，「うどんをやたら多く見出す世界探索の集合」であることに変わりはないとしてしまえる ^原注20^。 （ 定延 2001: 134，下線は筆者）

### 2.2 佐藤 (2017) の指摘

これに対し， 佐藤 (2017) は「探索の領域が〔筆者略〕探索という行動の集合である場合に，多少は印象的・感覚的であってもよいという説明に，妥当性はあるのだろうか」（ 佐藤 2017: 7）と疑問を呈し，「ばかり」と非該当例の関係について， 定延 (2001) とは異なる議論を展開している。

佐藤 (2017) は，「認識的際立ち性」という観点から「ばかり」の振る舞いを説明している。 佐藤 (2017) によれば，「認識的際立ち性」とは次のようなものである。

(13) ここ〔筆者注: 佐藤 (2017)〕で言う「認識的際立ち性」とは，当該の主体にとって何らかの意味において容易に捉えられるもの，捉えずにはいられない際立ちをもつものである。 （ 佐藤 2017: 9）

佐藤 (2017) は，集合を問題にする言語形式には，予め確立されている客観的な集合だけでなく，話者の経験に根差して形成された主観的な集合に関与するものがあると述べ（ 佐藤 2017: 8），その一例として「ばかり」を挙げている。また，後者の集合が形成されるに当たっては様々な動機があり得るとしており（ 佐藤 2017: 4-5），特に「ばかり」が関与する集合が形成される動機となるのが「認識的際立ち性」であると指摘している（ 佐藤 2017: 9）。

佐藤 (2017) によれば，「ばかり」が用いられるに当たっては，「認識的際立ち性という動機づけに支えられ，その特徴を有する事態のみを成員とする経験記憶の集合が形成される」（ 佐藤 2017: 9）とされる。例えば， 佐藤 (2017) は次の (14) の文が発話されるに至る過程を (15) のようにまとめている。

(14) あなた，学校に遅刻して ばかりでどうするの （ 佐藤 2017: 3，傍点を下線に改変）
(15) 「ばかり」の集合形成の事例②
  a. 母親が娘の登校時間を気にしながら日常生活を送る。
  b. 週 2 回のペースで娘の学校への遅刻という認識的際立ち性を有する事態を知覚する。
  c. 「娘の遅刻」という認識的際立ち性を有する事態のみから成る経験記憶の集合が形成され，「遅刻してばかり」という認識にいたる。 （ 佐藤 2017: 10，下線は筆者）

仮に，週 6 日制の学校に「週 2 回のペース」で遅刻した場合，週 4 回は遅刻していないことになり，(14) の文においてはそれが非該当例となる。しかし，「認識的際立ち性」という特徴を持つもので構成される主観的な集合には「遅刻」のみが含まれる，言い換えれば「非遅刻」は含まれないため ^10^，(14) の文が問題なく成立するとされるのである。

### 2.3　本稿の主眼

以上， 定延 (2001) と 佐藤 (2017) の議論を概観した。いずれにおいても「ばかり」が非該当例の関係について興味深い指摘が見られるが， 佐藤 (2017) も述べているように， 定延 (2001) の指摘には検討の余地がある。これを踏まえ，本稿では「ばかり」と非該当例の関係について， 佐藤 (2017) の考えを採る ^11^。

一方で，両者の関係については従来考察の対象とされていない問題がある。次の文はいずれも「ばかり」を含むため，先行研究に倣えば非該当例（「赤いシャツ」「黒猫」）が存在していても成立することが予測されるが，(16b) (17b) については成立しない。

(16) 【白いシャツを 900 枚，赤いシャツを 100 枚購入した場合】
  a. 白いシャツ ばかりを購入した。 (=(2a))
  b. # 白いシャツ ばかりを 1000 枚購入した。 (=(4))
(17) 【三毛猫が 40 匹，黒猫が 10 匹集まった場合】
  a. 三毛猫 ばかりが集まった。 (=(3a))
  b. # 三毛猫 ばかりが 50 匹集まった。 (=(5))

(16a) (17a) と (16b) (17b) の相違点は，後者には数量詞が生起しているという点である。

これは数量詞が示す事物の数量の中において非該当例の存在が許容されないということを示しており，先行研究で言われる非該当例の位置づけについて重要な意味を持つ。ただし，この現象は遊離数量詞が出現する場合には明確であるが，(18b) のように非遊離数量詞（名詞句内の数量詞）が出現する場合には解釈がやや曖昧になるようである。

(18) 【女性を 400 人，男性を 100 人招待した場合】
  a. # 女性 ばかり 500人招待した。
  b. 招待した 500 人は女性 ばかりだ ^12^。

つまり，これらの現象は次のことを示していると言える。

(19) 「ばかり」が遊離数量詞と共起する場合，当該の数量詞が示す事物の数量の中において非該当例の存在が許容されない。

前述の通り，先行研究では「ばかり」が非該当例を許容する（ように見える）ことやその要因については指摘されてきたが， (19) のような現象について指摘・考察した研究は管見の限り存在しない。従って，本稿ではこの (19) の現象に注目し，改めて「ばかり」の意味について考察する。

## 3.　「ばかり」と遊離数量詞

はじめに，「ばかり」と遊離数量詞の関係について考察する。以下では，先行研究の指摘を参考にし，「ばかり」が設定する集合の特徴，及び遊離数量詞の特徴について確認する。

### 3.1　「ばかり」が設定する集合と事態の数量

まず，「ばかり」が設定する集合の特徴について， 佐藤 (2017) の指摘を踏まえて確認する。前述の通り， 佐藤 (2017) は，「ばかり」は「認識的際立ち性」という特徴を持つもののみで構成される主観的な集合を設定するとしているが (2.2 節)，その点について次のように述べている。

(20) 認識的際立ち性という動機づけに支えられ，その特徴を有する 事態のみを成員とする経験記憶の集合が形成される。 （ 佐藤 2017: 9，下線は筆者）

また， 佐藤 (2017) は「認識的際立ち性」が生じる要因の 1 つとして次の (21a) を挙げ，これについて (21b) のように述べている。

(21) a. 知覚経験される事態の数が 多い。最低でも複数である。 （ 佐藤 2017: 11，下線は筆者）
b. 一度の失敗しか経験されていない場合，「失敗ばかり」とは言えないだろう。したがって，この要因は 「ばかり」が使われるための必要条件である ^原注6^。 （ 佐藤 2017: 11，下線は筆者）

このように， 佐藤 (2017) は「ばかり」が設定する主観的な集合に含まれるのは「事態」であり ^13^，その数が「多い」ことが「ばかり」が用いられる要件であると指摘している。

次に，この指摘を踏まえつつ，「ばかり」が非該当例を許容する（ように見える）背景について改めて検討する。前述の通り，次の (22) の文は (23) の状況において問題なく成立する。

(22) 白いシャツ ばかりを購入した。
(23) 白いシャツを 900 枚，赤いシャツを 100 枚，計 1000 枚のシャツを購入した。
  このとき，「ばかり」が設定する集合に含まれるのは「事態」であるということを踏まえると， (22) の文が成立するに当たり， (23) の状況は次のように捉え直されていると考えられる。
(23’) 「白いシャツを購入する」という事態が 900，「赤いシャツを購入する」という事態が 100， 計 1000 の「購入する」という事態が生じた。

(22) の文が成立するということは，事態の総数は 1000 であるものの，「ばかり」はそのうちの（「認識的際立ち性」を持つ）900 の事態のみから成る集合を設定し得るということになる。このとき， (22) では「白いシャツを購入する」という事態の数が多いということは間接的に表現され得るが ^14^，その具体的な数（総数に一致する数なのか，あるいはそれに近い数なのかということ）には関与していない。つまり，「ばかり」は次のような特徴を持つと言える。

(24) 「ばかり」は事態から成る主観的な集合を設定するが，その事態の具体的な数には関与しない。

### 3.2　遊離数量詞と事態の数量

次に，遊離数量詞に関する先行研究の指摘を見る。 矢澤 (1985) は，本稿での遊離数量詞に当たる「NCQ型」の数量詞 ^15^について，「何らかの形で動詞の表す動作・作用に関連した数量を表しているのではないか」（ 矢澤 1985: 104）と述べ ^16^，「NCQ型」の数量詞とそれ以外の数量詞の相違点について次のように指摘している。

(25) NCQ 型の数量詞〔筆者注:本稿での遊離数量詞〕は，述部に直接関わり，その述部の表す動作・作用の上で先行名詞句と間接的な意味的関係を結ぶのに対し，NCQ 型以外の型の数量詞は，先行名詞句に直接関わり，先行名詞句が述部と関わることによって，数量詞と述部との間接的な関係ができると考えるのである。 （ 矢澤 1985: 105-106，下線は筆者）

この指摘は，遊離数量詞が事態と密接に関わることを示している。具体的には，遊離数量詞は次のような特徴を持つと言える。

(26) 遊離数量詞は，事態の数量を表す（数量を事態の数量として表し直す）。 (=(6))

### 3.3　「ばかり」と遊離数量詞の関係

以上の点を踏まえ，「ばかり」と遊離数量詞の関係について考察する。 前述の通り，次の (27) (28) の場合，遊離数量詞を含まない (27a) (28a) は成立するのに対し，それを含む (27b) (28b) は成立しない。

(27) 【白いシャツを 900 枚，赤いシャツを 100 枚購入した場合】
  a. 白いシャツ ばかりを購入した。 (=(2a)(16a))
  b. # 白いシャツ ばかりを 1000 枚購入した。 (=(4)(16b))
(28) 【三毛猫が 40 匹，黒猫が 10 匹集まった場合】
  a. 三毛猫 ばかりが集まった。 (=(3a)(17a))
  b. # 三毛猫 ばかりが 50 匹集まった。 (=(5)(17b))

3.1 節で述べた通り，当該の場合に (27a) (28a) が成立するのは，「ばかり」はそれが設定する主観的な集合に含まれる事態の具体的な数には関与しない ((24)) ためである。それにもかかわらず，同様に「ばかり」を含む (27b) (28b) が成立しないということは，遊離数量詞が共起することで，その事態の数が具体的に定められることによると考えられる。つまり，(27b) (28b) では，「ばかり」が設定する主観的な集合に含まれる「白いシャツを購入する」「三毛猫が集まる」という事態の数量が「1000」「50」であるということが表されると言える。このことは次のようにまとめられる。

(29) 「ばかり」が数量詞と共起する場合，当該の数量詞は客観的な集合ではなく，「ばかり」が設定する主観的な集合に含まれる事態の数量を表す。 (=(6b))

## 4.　「ばかり」と「限定」

次に，3 節の観察を踏まえて「ばかり」と「限定」の関係について考察する。以下では，まず，「ばかり」と非該当例の問題に触れる先行研究のうち，「ばかり」と「限定」の関係にも言及するものとして 日本語記述文法研究会 (2009) と 澤田 (2007) の指摘を概観する。その上で，本稿の主張を述べる。

### 4.1 日本語記述文法研究会 (2009) の指摘について

まず， 日本語記述文法研究会 (2009) は次のように述べ，「ばかり」は「限定」を表すと主張している ^17^。

(30) 「ばかり」は， とりたてた要素が唯一のものであることを示し，ほかのものを排除するという 限定の意味を表す。（ 日本語記述文法研究会 2009: 61，下線は筆者）

また， 日本語記述文法研究会 (2009) は次の (31) の文について (32) のように述べ，「ばかり」と非該当例の関係に触れている。

(31) 佐藤さんは来客にコーヒー ばかり出した。 （ 日本語記述文法研究会 2009: 62）

(32) コーヒー以外のものも出した可能性は完全には否定されない。 （ 日本語記述文法研究会 2009: 62）

さらに， 日本語記述文法研究会 (2009) によれば，「ばかり」が表す限定には次の 2 つの下位分類があり，(31) のような場合は (33b) の「限定の仕方」が採られているとされる。

(33) a. とりたてた要素が唯一のものであることを示し，ほかのものを排除するという限定 の仕方 （ 日本語記述文法研究会 2009: 62）
b. とりたてた要素が関わる事態が 何度も繰り返されることや，とりたてた要素が重なって 多数にのぼることを表すという限定の仕方（ 日本語記述文法研究会 2009: 62，下線は筆者）

日本語記述文法研究会 (2009) の指摘は，非該当例の問題について「限定」という意味の下で説明しようと試みている。しかし，その説明には不十分な点がある。確かに，(31) の文は「コーヒーを出す」という事態が複数回生じていなければ成立せず，その点で (33b) において述べられているように「何度も繰り返されること」「多数にのぼること」を表していると言える。しかし，その (33b) を (30) の下位分類としていることには問題がある。具体的に言えば，「何度も繰り返されること」「多数にのぼること」 ((33b)) と「唯一のものである」「ほかのものを排除する」 ((30)) ということには隔たりがある。それにもかかわらず， 日本語記述文法研究会 (2009) ではその点について特段の言及がなされていない。この点に鑑みれば， 日本語記述文法研究会 (2009) の説明は十分とは言えない ^18^。

### 4.2 澤田 (2007) の指摘について

次に， 澤田 (2007) は 菊地 (1983) が挙げる次の (34) の文について (35) のように述べている。

(34) この一週間そば バカリ食べたよ。 （ 菊地 1983: 58，下線は筆者）
(35) 「ばかり」を使用する第一の目的は，「毎日そばを食べた」とカテゴリーを限定するというより， 話し手が「この一週間を思い起こせば，よくそばを食べた，それは通常 の一週間より多すぎた」ということを伝える方が重要であり，その 二次的な効果として明示された要素に対比される要素（明示された要素以外にその現象を成り立たせる可能性のある要素)がその観察された中に少なかった。または，なかったと伝えることになる。 派生的に，限定的解釈がでてくるのである。 （ 澤田 2007: 118-119，下線は筆者）

このように， 澤田 (2007) は，「ばかり」は「通常より多い」ということを表すのであり，「限定（的解釈）」はそこから「派生」する「二次的な効果」であると捉えている。

ここで注目したいのは，「ばかり」が設定する集合と非該当例の関係である。(35) では，「ばかり」は「明示された要素に対比される要素」が「観察された中に少なかった」「または，なかった」ということを伝えるとされている。しかし，「ばかり」が設定する集合との関係を考えた場合，当該の要素がその集合の内部に存在するのか外部に存在するのかという点において，この指摘は曖昧である。これに対し，本稿では，非該当例は「ばかり」が設定する集合の内部には存在しないと考える。それは次の現象が示唆している。

(36) 「ばかり」が遊離数量詞と共起する場合，当該の数量詞が示す事物の数量の中において非該当例の存在が許容されない。(=(19))

前述の通り，「ばかり」が遊離数量詞と共起する場合，その数量詞は「ばかり」が設定する主観的な集合に含まれる事態の数量を表すが ((29))，その集合の内部に非該当例が存在し得るのであれば，次の文も，場合によっては「1000」の「購入する」という事態の中に非該当例（「赤いシャツを購入する」）が含まれていても成立するということになるが，次の文がそうした状況下では成立しないことは前述の通りである。

(37) 【白いシャツを900枚，赤いシャツを100枚購入した場合】
# 白いシャツ ばかりを 1000 枚購入した。 (=(4)(16b)(27b))

### 4.3　本稿の主張

以上を踏まえ，ここで本稿における「ばかり」と「限定」，「ばかり」と非該当例の関係について， 日本語記述文法研究会 (2009)， 澤田 (2007) との違いを明確にした形で述べる。本稿の主張は次の通りである。

(38) とりたて詞「ばかり」は「限定」を表し，非該当例は「ばかり」が問題にする集合の外
部においてのみその存在が許容され得る。 (=(7))

本稿では，「ばかり」は「限定」，即ちある集合の内部において，とりたて詞がとりたてる要素が存在し，それ以外の要素（非該当例）が存在しない ((1)) ことを表すと主張する。ただし，その「限定」は「認識的際立ち性」などに起因して形成される主観的な集合の内部に対してのものである。従って，客観的な集合の内部に非該当例が存在していても，それが（「認識的際立ち性」を持たないが故に）「ばかり」が設定する主観的な集合に含まれなければ，「ばかり」は用いられ得る。つまり，本稿の主張で言えば，非該当例は「ばかり」が設定する主観的な集合の外部に，言わば「ばかり」が言及・関知しない存在として許容されるということになる。

本稿の主張は 日本語記述文法研究会 (2009) と同様に「ばかり」における非該当例の問題を「限定」という枠組みの中で説明するものであるが， 日本語記述文法研究会 (2009) が「ばかり」による「限定」を2つのタイプに分けた上で説明を試みているのに対し，本稿は「限定」のタイプを分けることはせず，関係する集合を2つに分けるという点で異なる。

また，本稿はとりたてる要素が「多い」場合に「ばかり」が用いられる（用いられやすい）と考える点で 澤田 (2007) と一致している （3.1 節）。しかし， 澤田 (2007) は「多い」ということを伝えるのが「重要」であり，「限定（的解釈）」は「二次的」と位置づけているのに対し，本稿は「限定」が「二次的」とは捉えず，また，「多い」というのは「ばかり」が集合を設定するに当たっての前提条件であると考える点で異なる。

以上，本稿の立場は「限定」の定義を1つに絞ることができる点，先行研究においてやや曖昧であった「限定」に関わる集合に対する非該当例の位置づけを相対的に明確にできる点でメリットがあると考える。

## 5.　おわりに

本稿では，「ばかり」が遊離数量詞と共起する場合に当該の数量詞が示す事物の数量の中において非該当例の存在が許容されない現象に注目し，遊離数量詞が「ばかり」によって設定される主観的な集合に含まれる事態の数量を表すということを明らかにした上で，「ばかり」は「限定」を表すということを主張した。

また，本稿では， 佐藤 (2017) の指摘を踏まえ，「ばかり」が問題にするのは世界を反映する予め確立された客観的な集合ではなく，自己の経験に根差して形成される主観的な集合であると捉えることにより，「限定」という意味の下で非該当例の問題が説明されると論じた。これは，「ばかり」の意味記述においては，その意味の対象となる集合（以下，対象集合）が重要となることを示しているが，この対象集合という視点の有用性は，「ばかり」の意味記述に限られるものではないと考える。まず挙げられるのは，他のとりたて詞の意味記述に当たっての有用性である。管見の限り，従来のとりたて詞研究では，とりたて詞各語について対象集合が詳細に議論されることや，それぞれの対象集合の設定のされ方の異同を本格的に取り上げた考察はほとんど行われていない。他のとりたて詞についても対象集合に関する考察を深めることで，個別のとりたて詞の意味やとりたて詞全体の意味体系の記述の精緻化が可能となろう。また，とりたて詞に留まらず，非該当例を許容しないとされる諸形式の意味記述に当たってもこの視点が有用であると考えられる。例えば，全称量化詞などと呼ばれる「全部」「みんな」，さらに「常に」「いつも」などは，基本的には非該当例を許容しないとされるが，「みんな」や「いつも」など一部の形式については非該当例を許容し得る。このこと自体は既に 佐藤 (2017) で指摘されており，意味的な観点からその要因を明らかにしようとする研究も存在する（ 大塚 2020， 2021）。しかし，対象集合に注目して再検討することで，先行研究において未だ解明されていない点について説明を与えることが可能になると考える。これらについては稿を改めて論じることとする。

### データ可用性

本論文の研究結果の基礎となるデータは，すべて本論文中に示されており，追加のソースデータは必要とされていない。例外として，注4で示したデータ絞り込みの結果，判断を加えた 42 例の提示は，国立国語研究所による現代日本語書き言葉均衡コーパス (BCCWJ) 「中納言」より入手できる。同コーパス利用には登録が必要だが，他の研究者も著者と同じようにデータにアクセスできる。登録方法については https://chunagon.ninjal.ac.jp/auth/login?service=https%3A%2F%2Fchunagon.ninjal.ac.jp%2Fj_spring_cas_security_check を参照されたい。

### 謝辞

本稿は，国際研究集会「次世代の日本研究―国際的協働研究と研究交流―」（2021 年 3 月 21 日，オンライン）における口頭発表の内容に加筆・修正を施したものである。発表に際し，貴重なご意見を賜った方々に感謝申し上げる。

### V2における著者所属変更について

大塚貴史　筑波大学からの異動のため

大東文化大学 外国語学部 日本語学科

Department of Japanese Language, Faculty of Foreign Languages, Daito Bunka University, Itabashi-ku, Tokyo, 175-8571, Japan

白川稜　筑波大学大学院修了のため

愛国学園大学 人間文化学部（非常勤）

Faculty of Human and Cultural Sciences, Aikoku Gakuen University, Yotsukaido, Chiba, 284-0005, Japan

橋本修と沼田善子の所属に変更はない。

## Footnotes

^1^
(1) の規定は主に 沼田 (2009) に基づく。 沼田 (2009) は，「とりたて詞がとりたてる文中の要素」（ 沼田 2009: 37）を「自者」，それに「端的に対比される『自者』以外の要素」（ 沼田 2009: 37）を「他者」とし，「自者」が肯定され，かつ「他者」が否定されることを「限定」と呼んでいる（ 沼田 2009: 196）。

^2^
先行研究から引用した例文などの末尾にはその出典を記す。一方，出典のないものは筆者によるものであるが，筆者の作例には「#」を付すことがある。これは，当該の文が文法的ではあるものの，指定の文脈では不自然ということを示す記号である。また，引用した例文には「?」「??」を付すことがあるが，これは引用元の文献に倣ったものであり，いずれも当該の文が （やや）不自然であることを示す記号である。

^3^
「非該当例」という用語は 佐藤 (2017) に倣ったものである。なお， 2.1 節で触れる 定延 (2001) は「夾雑物」という用語を用いているが，煩雑化を避けるため，本稿では「非該当例」に統一する。

^4^
先行研究では，数量詞の捉え方について幾つかの立場があり，遊離数量詞と呼称すべき範囲，あるいは名称そのものについても議論がある（詳細は 矢澤 (1988) や 加藤 (1997) などを参照されたい）。しかし，本稿ではその点には立ち入らず，副詞位置に生起する数量詞を便宜的に遊離数量詞と呼称する。

^5^
(4) (5) は作例であるが，コーパスにおいても「ばかり」に数量詞が後続した文が存在し，これらについての日本語母語話者の内省判断において， (4) (5) と同様に遊離数量詞が示す数量の中に非該当例の存在が許容されないことを確認している。なお，コーパスは『現代日本語書き言葉均衡コーパス』 (BCCWJ) を使用した。該当文抽出の手順，及び内省判断の手順は以下の通りである。 ・BCCWJ をコーパス検索アプリケーション「中納言」で使用 ・検索と抽出の手順は，  短単位検索  キー: ばかり  後方共起:キーから 1 語，品詞の小分類が名詞・数詞  →ヒット数 45 例  →上記 45 例を目視で確認，バグ 3 例を除外  → 残った 42 例について日本語母語話者により内省判断 以上の手順により抽出された文例を 1 例示す。  (i) 今日は，映画の予告編ばかり二十四本見てきました。 （サンプル ID: OY15_13680，yahoo! 知恵袋）

^6^
(4) (5) について，非該当例の存在が認められる場合でも成立すると判断する話者の存在も完全には否定できない。ただし，本稿においてこれらが当該の文脈で成立しないと主張するのは意味論のレベルであるのに対し，成立するという判断は語用論のレベルでなされるものであると考える。語用論の 1 つのモデルである「関連性理論」 (Relevance Theory) を提唱する Sperber and Wilson (1995) は，「思考の最適な解釈的表現は，聞き手にその思考について処理するに値するだけの関連性がある情報を与え，できるだけ処理労力が少なくてすむようにしなくてはなら」 ( Sperber and Wilson 1995: 284) ず，「厳密に言えば偽とわかっている」 ( Sperber and Wilson 1995: 284) 内容でも成立する場合があるとしている。非該当例が認められる場合でも (4) (5) が成立するという判断があり得るとすれば，それはこうした語用論のレベルでの判断であり，本稿が目的とする意味論のレベルの議論とは区別されるべきものである。

^7^
BCCWJ からの用例の抽出方法は注 5 の通り。

^8^
「 『ばかり』が設定する集合」とは，沼田 (2009) 等で述べる，「自者」とそれに対する同類の「他者」が構成する集合である。詳しくは 沼田 (2009: 43-56) を参照されたい。

^9^
「探索領域」は「探索が及ぶ領域」（ 定延 2001: 118）を，「探索課題」は「探索者が探索を通して解決しようとする課題」（ 定延 2001: 119）を意味する。

^10^

佐藤 (2017) は，「認識的際立ち性という性質をよりもちやすくする要因」（ 佐藤 2017: 11）の 1 つとして次のことを挙げている。

(ii) 事態が信念に照らし合わせて 有標的である。 （ 佐藤 2017: 11，下線は筆者）

この指摘は，次のような文の容認度の差が踏まえられている。

(iii) 太郎は授業を さぼってばかりだ。 （ 佐藤 2017: 12，下線は筆者）
(iv) ??太郎は授業に 出席してばかりだ。 （ 佐藤 2017: 12，下線は筆者）

佐藤 (2017) は，「常識的な信念を有するものにとって，『授業をさぼる』〔筆者略〕といった行為はあるまじきものであり，有標性の高いものといえよう」（ 佐藤 2017: 12）と述べている。そのために「認識的際立ち性」が生じやすく， (iii) は自然な文となる。一方， (iv) が不自然なのは，「授業に出席す」という事態は「有標性」が低く，「認識的際立ち性」を持ちにくいためであると推察される。「非遅刻」という事態が「ばかり」が問題にする集合に含まれないのも，この事態が「授業に出席する」という事態と同様に「有標性」が低いためであると考えられる。

^11^
なお， 佐藤 (2017) は「本稿〔筆者注: 佐藤 (2017) 〕が論じた集合形成の議論における知覚経験という観点は， 定延 (2001) の言うところの『探索』というわれわれの心身の行動を前提とするものであり，その意味で本研究は 定延 (2001) の議論の延長線上に位置づけられる」（ 佐藤 2017: 13）と述べており， 定延 (2001) が提唱する「探索」という行動そのものに異議を唱えているわけではない。これについては本稿も同様である。

^12^
(18b) についても当該の場面では成立しないと判断する話者も存在するようであるが，少なくとも相対的には (18b) の方が文脈的自然度は高いと考えられる。

^13^
「ばかり」と事態の関わりについては 佐藤 (2017) 以前にも示唆・指摘されている。例えば， 森田 (1980) は次の (v) のように， 菊地 (1983) は (vi) のように述べている。

(v) 「ばかり」は，“ある同一同類の主体がある範囲で 行う”とか，“同一同類の事柄をある範囲内で 行う”とか，“同一同類の対象に対して 行われる”とか，また，“ある同一の事物がある範囲の程度内で 存在する”とか，あるいは“ある事態に 対応するのがいつも同じ人物である”とか，いずれの場合も 動詞的叙述(傍点部分〔筆者注:本稿中下線〕)を前提としている。 （ 森田 1980: 402，下線は筆者）
(vi) バカリは，<同類として括れる 事態が数多くみとめられる>時に使われる。 （ 菊地 1983: 57，下線は筆者)

なお， 定延 (2001) は 菊地 (1983) による(vi)の指摘に触れた上で，「 『ばかり』の探索領域が事物の集合ではなく，事物を探索領域とする探索の集合であると考える点で，本稿〔筆者注: 定延 (2001) 〕は菊地〔筆者注: 菊地 (1983) 〕と同じ立場に立つ」 （ 定延 2001: 130）と述べている。その点では， 定延 (2001) も「ばかり」は事態に関わると捉えていると言える。

^14^
事態の数が多いということは「ばかり」が用いられる動機となり得る ((21)) というだけで，「ばかり」が直接的に表現しようとする内容ではない。ただし，それに起因して「ばかり」が用いられることがある以上，間接的には「ばかり」は事態の数が多いということを表し得ると言える。

^15^

奥津 (1983) 以降の数量詞研究では，しばしば「 NCQ 型」「NQC 型」「NノQC 型」「QノNC 型」といった名称が用いられる。これらの名称は，数量詞をその現れ方によって分類した際に用いられるものであり，Nが名詞を，Cが格助詞を，Qが数量詞を指している。

^16^
この 矢澤 (1985) の指摘は，「NCQ 型の数量詞は，述部が動詞句以外のときには，現れにくいという構文上の制約がある」（ 矢澤1985: 103）ことに基づいている。 矢澤 (1985) は，述部が動詞句以外である次の文において，「NCQ 型」の数量詞を含む (vii) (viii) (ix) とそれ以外の数量詞を含む (x) (xi) (xii) では，前者の方が容認度が低いことを示している。

(vii) ?ココニイル女性ハ 五人 高校生ノ先生ダ （ 矢澤 1985: 103）
(viii) ?アノ会社ノ受付嬢ハ 三人 美シイ （ 矢澤 1985: 103）
(ix) ?アノ台ノ上ニ並ンダ牛乳ハ 五本 古イ （ 矢澤 1985: 103）
(x) ココニイル女性 五人ハ 高校生ノ先生ダ （NQC 型） （ 矢澤 1985: 103）
(xi) アノ会社ノ受付嬢ノ（中ノ） 三人ハ 美シイ （NノQC 型） （ 矢澤 1985: 104）
(xii) アノ台ノ上ニ並ンダ 五本ノ牛乳ハ 古イ （Qノ NC 型） （ 矢澤 1985: 104）

^17^
「ばかり」が「限定」を表すということについては多くの先行研究で指摘されている（ 丹羽 1992; 益岡・田窪 1992; 中西 1995; 安部 2001; 沼田 2009 など）。

^18^
なお， 2 つ提示されている「ばかり」の「限定の仕方」の1つである (33a) の説明は，その上位に当たる「限定」の意味に関する (30) の説明と完全に一致しているが，これはそもそも下位分類の設定として適切とは言い難い。この点も， 日本語記述文法研究会 (2009) の捉え方に検討の余地があることを示唆している。
