# Supplementary material for: “Exclusivity” and quantifier float in&nbsp;<i>bakari</i>
Source: F1000Res. 2022 Jul 4;10:1276. [Article in Japanese] Originally published 2021 Dec 14. [Version 2] doi: 10.12688/f1000research.55582.2 (PMC9277704; doi:10.12688/f1000research.55582.2)
Supplement: Peer review history

# Peer review: Reviewer response for version 2

**DOI:** 10.5256/f1000research.134652.r142986

**Published:** 2022-08-16

**Authors:** Yasuto Kikuchi

**Affiliations:** 1 Faculty of Letters, Kokugakuin University, Tokyo, Japan

１　主な論点の確認

 前稿については、査読者の査読に対して､著者の一人からの回答中に、著者の趣旨を査読者が正しく理解していない旨の指摘が（わかりにくい書き方をしてしまったとの「お詫び」とともに）あったので、今回の稿については、改めて注意して読んだつもりである。

 まず、本稿の主な論点についての査読者の理解を、査読者の言葉でできるだけ平易に述べて確認することから始める。

 1）バカリは一般に「非該当例」を許容する（先行諸研究の指摘の通り）。

 2）バカリが「非該当例」を許容する理由／原理については、定延（2001）と佐藤（2017）にそれぞれ説明がある。本稿は佐藤の説明に従う。佐藤の説明の中核的な内容は、バカリは、話者の経験に基づいて〈認識的際立ち性〉を有する事態の主観的な集合を形成して「当該の事態バカリ」と捉えるものなので、客観的事態と比べれば「非該当例」が生じる、というものである。

 3）遊離数量詞と共起するバカリは「非該当例」を許容しない（本稿の発見）。

 4）本稿は、3)の理由を、次のようなルールによると考える。

 「バカリと遊離数量詞が共起するとすれば、遊離数量詞は〈バカリの設定する集合（佐藤のいう主観的な集合）の事態の数〉を表すものとしてのみ現れうる。バカリと共起する遊離数量詞が〈客観的な集合の事態の数〉を表すことはできない。」（本稿(29)(36)と同趣旨。述べ方を少し変えた。こちらのほうがわかりやすいのではないか。）

 5）4)は、2)で見た、遊離数量詞を伴わない場合のバカリの性質と符合する。

 6）遊離数量詞を含まない場合と含む場合とでは、「非該当例」の許容について、1) 3)のような相違があるが、5)の意味で、遊離数量詞のある場合のバカリが ことのほか特別な振る舞いをしていると見る必要はなく、遊離数量詞の有無にかかわらず、バカリ自体は一元的に捉え得ると考える。

 ７）６)のように見れば、遊離数量詞の有無にかかわらず（＝「非該当例」の不許容・許容にかかわらず）、バカリの意味を「限定」と見てよいと考える。

 といったところであろうか。４)５)６)が、前稿時点の述べ方では、査読者には明瞭に見てとれなかった点である。

 ２　評価できる点、支持できる点

 ・最も評価できる点は、前回も記したが、上記３)である。

 ・上記１)２)も、内容的に適正であるが、ともに先行研究に負う点である。

 ・上記４)５)６)は、本稿の見方であるが、おそらく支持してよいと思われる。

 ３　問題点

 次の３点が指摘できる。

 ［Ⅰ］バカリの意味を「限定」と見ることについて。上記６)まではよいとしても、６)から７)への運びが粗雑である。バカリの意味（名づけ）として「限定」がふさわしいという論証は、できていない（と、査読者には判断される）。

 ［Ⅱ］上記４)（本稿(29)）について。もう少し論述の補強が必要。

 ［Ⅲ］「主観的集合」と「客観的集合」について。対照的な名づけをしているが、両者はかなり異質である。この点につき、もう少し確認しながら論述を進めてほしい。

 問題点の大きさとしては、［Ⅰ］が最も大きく、次いで［Ⅱ］［Ⅲ］の順である。以下では、逆順に見ていく。以下、４で［Ⅲ］、５で［Ⅱ］、６で［Ⅰ］を見る。

 ４　「主観的集合」と「客観的集合」について　［問題点Ⅲ］

 上記１の項で４）としてまとめた内容（本稿(29)(36)）は、「主観的集合」と「客観的集合」を区別することで、遊離数量詞がある場合に「非該当例」が許容されないことを説明しようとしたものだが－この問題を解くための説明としては、これはぎりぎり認めてよいものかと思われ、上記２では「おそらく支持してよい」としたが－、「主観的集合」と「客観的集合」は、かなり異質のものと見られ、このことについて、簡単な対照的説明がほしい（と、査読者は感じた）。

 前者は、話者の経験に基づいて〈認識的際立ち性〉を有する事態の主観的な集合が形成されるとするもので、このような集合形成がバカリを特徴づけるものである。集合の要素は事態であり、形成の仕方は、普通の意味での集合の場合とは少し異なるものである。一方、後者は、……というような説明である。現状のままだと、特に「客観的集合」についての説明に不足感があり、ちょっとわかりにくかった。

 あるいは、「客観的集合」というものを持ち出さずに論を展開する可能性を探ってはどうか、という気がした。

 ５　本稿(29)にかかわる論述の、さらなる補強について　［問題点Ⅱ］

 ここにあげられている場合以外にも、(29)に関係して、読者から見て「このケースは、論者の立場ではどういう扱いになるのだろう」と気になる例が考えられる。それらについての補足説明をお願いしたい。

 まず、【白いシャツを1000枚買い，他のシャツは買わなかった場合】に

 　　白いシャツばかりを1000 枚購入した。

 と言えることに触れ、その説明をしてほしい。可能性としては「この場合は客観的な集合で、バカリを使いつつも、その数を正確に述べているので問題ない」といった説明もありえそうだが、論者の立場ではそうではなく、(29)のように主張することとの整合性から「この場合も、遊離数量詞は主観的な集合のあらわす事態の数を示している」といった説明になるのであろう。(29)のすぐ上の「つまり」で始まる文から、それは読み取れはするが、上のような文を例文として立てて、こうしたことを念のためはっきりと記してほしい。

 また、【白いシャツを1000枚買い，赤いシャツを10枚買った場合】に

 　　白いシャツばかりを1000 枚購入した。

 と言えるのかどうか（事実としてどうか）、それについてはどういう説明になるのかにも触れる必要があろう。逆からいえば、

 　　白いシャツばかりを1000 枚購入した。

 と言う場合、白いシャツは1000枚買う必要がある（1000枚の内訳の中に赤があってはいけない）が、白いシャツ1000枚の他に赤いシャツを少数だけ買った場合にもこの文は言えるのか、ということである。本稿の論者の立場からは、主観的な集合の「外部」になら非該当例の存在が許容されるということで、「言える」という予測になるのだろうと思われるが、この理解でよいか。また、事実としてその予測の通りなのかどうか。［この点について、査読者自身は自信をもっての判断はできない。ぎりぎり言えるようにも思うし、ちょっと苦しいようにも思う。個人差もあろうから、アンケート調査も行っていただきたいところである。］

 仮に「言える」が優勢なら、議論は強化される。一方、「言えない」が優勢だとしたら、「遊離数量詞がある場合は、主観的な集合の「外部」においても非該当例は許容されない」というように仮説を修正すべきことになる。ただ、もし、そうだとすると、遊離数量詞がある場合とない場合とでは、この点全くの大違いとなり、上記６)のような見方はできないことになってしまう。「内部」と「外部」を分けて見ることで、遊離数量詞の有無にかかわらない整合性を確保しようとしてきたストラテジーが「崩壊」することにもなり、結構な一大事ではある。ただ、だからといって、この問題に触れずに済ませるわけにはいかないように思われる。

 ６　バカリの意味を「限定」と捉えるのがふさわしい、という論証が成立していないこと［問題点Ⅰ］

 本稿を通じて、バカリの意味（名づけ）として「限定」がふさわしいという論証は、できていないと思われる。以下この点を詳しく述べ、（できることなら）著者の理解を得たい。

 バカリは、話者が〈認識的際立ち性〉を有する事態を主観的に集合として形成し、「当該の事態バカリ」と述べるものである、という趣旨の佐藤の論は好論であるが－本稿の著者もこの点を支持しているわけだが－、この論が、バカリの意味は「限定」である、という話にどう繫がるというのか。その説明が必要である。査読者は、かなり理解に苦しむところである。以下に見るように、おそらくその説明はできないのではないかと思われる。

 佐藤の論にもう一言付け加えるなら、そのような主観的集合に、集中的に（さらに強く言えば、専ら）関心を向けて述べるときにバカリを使う、というぐらいは言ってよいかと思う。ここまで述べると「限定」に少し繫がりそうな気もするが、なお考えると、やはり繫がりにくいように査読者には思われる。

 まず留意すべきは、「限定」というのは一般に、対象も基準・方法も客観的に捉えられる場合に使われるのが自然な語なのではないか、ということである（その意味でダケにはふさわしい）。バカリの場合、客観的には「限定」されていない。

 また、仮に「主観的な限定でも限定と言ってよいではないか」という立場をとるにしても、上記の佐藤の主張は、バカリは、その集合に集中的に関心を向けるときに使う語である、ということであって、これは、バカリが（主観的にであっても）「限定」する働きを持っている、というのとはかなり違う話である。「そこに専ら関心を向けた結果として限定のようなeffectが出る」という場合はあるにしても、それは「バカリそれ自体が、限定するという機能を本義として持っている」ということではない。

 肝心の佐藤も、また定延も、「限定」という語は全く使っていない。彼らの好論は、仮に「限定」という言葉を使って述べたとしたら、その最もよいところが伝わらなくなってしまうような論であるように、査読者には思われる。

 佐藤に（また定延にもある程度）拠りながら、最後に近いところで、十分な説明もなく「限定」を持ち込んで、「限定」を結論にしてしまうという論は、論の運びとして粗雑である。バカリは限定だとアプリオリに決めてかかっているという印象を受け、誠実な議論を尽くしていないといわざるを得ない。

 「限定」を持ち出すなら、澤田のように、基本義は別に立て、そこから派生的／二次的に「限定」の解釈が得られるとするのが順当で、これが精一杯のところであろう。

 念のため付言すると、助詞・助動詞の名づけは、その語の用法をあらわすのに最もふさわしい名を与えるべきである。「限定」は、日常語としては、一般に非該当例を許容しない意味で使うので、バカリを「限定」と呼ぶのはふさわしい名づけとは言いがたい（「学術用語としてはちょっと違ったこういう意味で使います」といったことを言い添えて普通のことばの使い方を大きく離れるのは、よいことではない）。加えて、非該当例を許さないダケの存在もあるので、バカリ・ダケをともに「限定」と呼ぶのは、どう説明を付け加えても混乱のもとであり、日本語学習者のためにもよくない。このように、「実態との乖離があり混乱を招く術語」を避けるのは、研究者の良心だと査読者は考える。（投稿者は、「本稿の立場は「限定」の定義を1つに絞ることができ……メリットがあると考える」（４節末尾）としているが、「１つに絞れた」かのように見えるのは、通常の意味では「限定」でないものを「限定」と呼んだからというだけのことであり、混乱を招くデメリットのほうが大きい。）

 なおまた、著者は、バカリを「限定」と見ることには多くの先行研究があるとして、それらを具体的にリストしており（注17）、このことを、バカリを「限定」と見る一つの根拠としているかのようにも読める。だが、これらの先行研究の多くは（全て？）、バカリの意味は「限定」と見るのがふさわしい、ということをきちんと論証してはいないと思われる。おそらく、初期のいくつかの研究が十分吟味をせず安易に「限定」の語を使い、以後の研究も安易にこの語を継承してきた、というだけなのではないか。そうして、すでに定延や佐藤の論が出た今、それ以前に「限定」を謳う先行研究がいかに多くあったとしても、その名づけは半ば価値を失っているというべきで、「だから「限定」と呼び続けてよい」とする論拠にはならないことに目を向けるべきである。

 実は、著者たちには、バカリが「限定」であるという信念（ビリーフ）が論点先取的に形成されているのではないか、このため、バカリが「限定」であると論証しようという姿勢もそもそもあまり強くなく、かつ、そのことに気づいていないのではないかという印象を、査読者は受けた。

 だが、本当に「限定」かを虚心坦懐に問い直した場合、「限定」だときちんと論証することは、すでに難しいのではないか。佐藤の好論を支えているのは、「限定」とはある意味で対極的な見方であることに気づくべきであろう。今後のバカリをめぐる研究動向・研究史は、「限定」ではないとする（あるいは，従来の「限定」とは一線を画した）見方が主流になっていくように、査読者には思われる。

 本論文を、「限定」のビリーフを唱え続ける論文とするか、「限定」を使わずにバカリを説く方向へと舵を切る論文とするか。著者の一人が多年この領域を牽引してきた研究者なだけに、今後の研究動向・研究史を見据える中で、熟慮の上、答を出していただきたい。査読者からの助言は突き詰めればただ１点、現状では「限定」と見る論拠は示し得ていないということである。

 ７　承認の可否について

 以上のような具合なので、問題点、特にⅠとⅡについて、できるだけ対応していただいた上での「承認」という判断になる。修訂をすすめたい箇所は他にもあり、以下に記しておく。

 ８　その他の問題点

 (１）要旨中の、次の書き方

 （原文）本稿では，「ばかり」が遊離数量詞と共起する場合に非該当例を許容しない現象の分析から，「ばかり」の意味を「限定」とすべきであると主張する。

 →　この書き方は、バカリが非該当例を許容しない「遊離数量詞と共起する場合」という特殊なケースを不当に一般化する議論を展開しているかのような印象を与える（前稿について査読者はそう読んでしまった）。書き方を再考されたい。

 (２)　ここでの予測通りには行かない例の扱い方（例(4)(5)についての注6）について（引用は省略）

 →「(4) (5) について，非該当例の存在が認められる場合でも成立すると判断する話者の存在も完全には否定できない。」という話が唐突に出てくる感がある。もう少し説明がほしい、ということが１つ。

 また、仮にそういう場合があったとして、注6の説明は妥当なのか。引用されている Sperber and Wilson の説明が当該のケースにあてはまるのかどうか、読者は判断できない（当該のケースについての説明も不足しているし）。現状のままでは、妥当性に疑問符がつくことは避けられない。

 仮にそういう場合があったとしても、その説明には、語用論など持ち出さなくても、「バカリは非該当例を許容する」といういわば基本ルールを（これは普通は遊離数量詞がある場合にはあてはまらないのだが）遊離数量詞がある場合にも拡大して適用（拡大して一般化）した［そういう人が一部いるのかも］、という説明でよいのではないか。こういうことはありうることで、注6の説明よりもずっと説得力があると思われる。

 (３)　第１節末で「本稿の主張」を予告する部分（(6)a，(6)b，(7)）（引用は省略）

 →　この段階でこういう「主張」を述べても、この段階での読者には、ほとんど理解できないので、意味はないように思う。この段階での「主張」の提示はせず、以下どのように論を展開していくかの予告（読者がそれなりにわかった気になるような予告）にとどめてはどうか。

 (４)　第２節冒頭等に見られる、「「ばかり」が非該当例を許容する（ように見える）」という書き方

 →　「（ように見える）」とわざわざ書き添えるのはなぜか。「許容しているように見えるが、実はそうではない」という考え方なのか。そのように考えるべきだという論拠は示されていないように思われる。現実に、非該当例を許容していると見て、それでよいのではないか。このように趣旨のわかりにくい「（ように見える）」の付け加えは、大変読みにくくなる。削除をすすめる。

 第２節末尾と3.1に見られる同様の書き方についても、削除をすすめる。

 (５)　2.1の(9)ｂの書き方

 （原文）「どういう検索なのか、1 探索ずつスキャニング探索」

 →　(9)ａに合わせて名詞で終える書き方にしたのだろうが、「どういう検索なのか」「1探索ずつ」という連用成分があって、これを受ける動詞がないという落ち着きの悪い文（非文）になってしまっている。(9)ａと不揃いにはなるが、「……１探索ずつスキャニング探索していく」（定延の原文はこうなっている）と動詞で終わるようにしてはどうか（または原文をいじって、「……どういう検索なのか という、１探索ずつ の……」とする）。

 (６)　2.3の冒頭から2つ目の文における助詞の誤記

 （原文）いずれにおいても「ばかり」 が非該当例の関係について……

 →　「が」では意味が通らない。「と」か？　

 (７)　2.3の後半　遊離数量詞以外の数量詞の場合に触れた箇所

 （原文）ただし、……。(18)

 →　この部分［＝(18)の前の「ただし、……」の一文と(18)］は，本文から外して注にすることをすすめる。この内容がここで本文に入ると、論の流れが妨げられ、読者にとっては理解が妨げられる。

 なお、(18)b「招待した 500 人は女性 ばかりだ」についても、(18)a「女性 ばかり 500人招待した」と同様、＃だというのが査読者の判断であるが（これは前回も述べた）、今回気づいたのだが、(18)bは遊離数量詞ではないものの、動詞を直接修飾する数量詞であり、そういうケースは遊離数量詞と同じように考えてよいのではないか。（つまり、「遊離」ということが大事なのではなく、「動詞に直接かかる」ということが大事なのでは。）

 (８)　3.1の(22)の直前の文

 （原文）次に，この指摘を踏まえつつ，「ばかり」が非該当例を許容する（ように見える）背景について改めて検討する。

 →「（ように見える）」については、上記(４)のところで述べた通り。

 もう１点、「背景」は、この文脈にふさわしい感じがしない。「背景」に代えて、「事情」ぐらいでいかがか。

 (９)　4.2中の澤田（2007）への論評

 （原文）このように， 澤田 (2007) は，「ばかり」は「通常より多い」ということを表すのであり，「限定（的解釈）」はそこから「派生」する「二次的な効果」であると捉えている。ここで注目したいのは，「ばかり」が設定する集合と非該当例の関係である。(35) では，「ばかり」は「明示された要素に対比される要素」が「観察された中に少なかった」「または，なかった」ということを伝えるとされている。しかし，「ばかり」が設定する集合との関係を考えた場合，当該の要素がその集合の内部に存在するのか外部に存在するのかという点において，この指摘は曖昧である。これに対し，本稿では，非該当例は「ばかり」が設定する集合の内部には存在しないと考える。

 →　２点、問題を感じる。

 １つは、「当該の要素がその集合の内部に存在するのか外部に存在するのかという点において，この指摘は曖昧である。」という述べ方。

 この「内部vs.外部」は、著者の問題意識に沿って構想された概念にすぎず、その区別にどのぐらいの意義があるのかは、現段階ではまだ見きわめきれていない。まして、澤田がこの論文を執筆した段階で問題になっていたわけでもなく、澤田がこれに触れていないのは無理もないことである。こうしたことについて、これに触れていないことがあたかも不備であるかのようなトーンが、「……曖昧である」という書き方には感じられる。もう少しフェアな述べ方ができないか。

 「これに対し，本稿では，非該当例は「ばかり」が設定する集合の内部には存在しないと考える」としているが、本稿のシステムからすれば、これは当然そうなる（非該当例は外部でなら存在が許容される、ということになる）であろう。正しいといえば正しいのだろうけれど、著者たちが当然そうなるシステムを作ったというだけのことで、澤田の不備を自分たちが改善したかのようにも響く書き方は、なじまない（印象が悪い）［と、査読者は感じる］。

 もう１つは、澤田の「通常より多い」は常に成り立つわけではなく、「圧倒的少数事例」を取りたてる場合もあることが、その後の佐藤により指摘されたわけなので、この点を修訂する注（例えば、「その後、バカリが「圧倒的少数事例」を取り立てる場合もあることが佐藤（2017）により指摘されたので、これを踏まえた修訂が必要であるが、ここでは澤田の原文のまま引く。」といった注）を、「通常より多い」のところに付けることが望ましい。（澤田の「多い」は4.3の終わりにも触れられているので、そこでも、この注を引いて「注〇参照」としておくほうがよいかもしれない。）　　　　　　　　　　　　　　　　　　以上

（該当する場合は要回答）統計分析および解釈は適切ですか。

対象外（統計を使っていない）

本研究は明確かつ正確に提示されたものであり、最新の文献を引用していますか。

はい

方法と分析について第三者による再現が可能となるよう十分な詳細が提示されていますか。

はい

結果の基礎となるソースデータはすべて入手可能で再現性を十全に保証していますか。

ソースデータは不要

結論は結果により妥当な裏付けを得ていますか。

一部該当

研究設計は適切で学術的価値がありますか。

一部該当

Reviewer Expertise:

日本語学、特に日本語文法。日本語教育。

I confirm that I have read this submission and believe that I have an appropriate level of expertise to confirm that it is of an acceptable scientific standard, however I have significant reservations, as outlined above.

# Peer review: Reviewer response for version 2

**DOI:** 10.5256/f1000research.134652.r142989

**Published:** 2022-07-25

**Authors:** Takuzo Sato

**Affiliations:** 1 Gakushuin Women's College, Tokyo, Japan

適切に改訂されているものと判断します。

（該当する場合は要回答）統計分析および解釈は適切ですか。

一部該当

本研究は明確かつ正確に提示されたものであり、最新の文献を引用していますか。

はい

方法と分析について第三者による再現が可能となるよう十分な詳細が提示されていますか。

はい

結果の基礎となるソースデータはすべて入手可能で再現性を十全に保証していますか。

はい

結論は結果により妥当な裏付けを得ていますか。

はい

研究設計は適切で学術的価値がありますか。

はい

Reviewer Expertise:

現代日本語の文法論および意味論

I confirm that I have read this submission and believe that I have an appropriate level of expertise to confirm that it is of an acceptable scientific standard.

# Peer review: Reviewer response for version 2

**DOI:** 10.5256/f1000research.134652.r142988

**Published:** 2022-07-21

**Authors:** Toshiyuki Sadanobu

**Affiliations:** 1 Faculty of Letters/Graduate School of Letters, Kyoto University, Kyoto, Japan

丁寧にリプライしてくださってありがとうございました。

 私が指摘した３つの問題点について、回答をいただき、対応していただきましたので、私としてはこれ以上の要求はありません。

 ただし、用語について、これでよいのかと感じる点が１点ありますので、そのことだけ、要望として書き添えておきます。

 要望というのは、「数量詞」という用語の用法についての表記です。

 投稿者の認識および措置は、次のようになっているようです。

 １．「数量詞」という用語に、広義の用法があることは承知している。

 ２．広義の用法の場合、「数量詞」には、数詞から構成されていない「山ほど」のような表現が含まれることも承知している。

 ３．数詞から構成されていない「山ほど」のような表現が、自説とうまく合わないことも認識している。

 ４．論文の中で「数量詞」という用語を、数詞から構成されていない「山ほど」のような表現を含まない、狭義で用いる。

 ５．数詞から構成されていない「山ほど」のような表現について、それが自説とうまく合わないということは、論文に書かない。

 ６．「数量詞」という用語を、数詞から構成されていない「山ほど」のような表現を含まない狭義で用いることについて、論文に書かない。

 以上の１～６のうち、少なくとも６、できれば５と６について、論文で明記していただくのが、読者にとって親切と思いますがどうでしょうか。

 「山ほど」のような表現が反例になるのではないのか？」「このような表現は考察対象に含まれないのか？」「そもそも著者は用語「数量詞」をどう意味で用いているのか？」といった、読者の要らざる疑問を生まないために、ということです。

 さらに、この措置をとることがアドホックなものではなく、数詞から構成される数量詞と「山ほど」のような表現との、本来的な違い（リプライに書いていただいたもの）に基づく、根拠を持ったものであることを書いていただくと、論文を読み進める読者の抵抗感はさらに減少するのではないかと思います。

 要望は以上です。この要望を受け入れてくださるか否かに関わらず、査読結果は承認で結構です。

（該当する場合は要回答）統計分析および解釈は適切ですか。

対象外（統計を使っていない）

本研究は明確かつ正確に提示されたものであり、最新の文献を引用していますか。

はい

方法と分析について第三者による再現が可能となるよう十分な詳細が提示されていますか。

はい

結果の基礎となるソースデータはすべて入手可能で再現性を十全に保証していますか。

はい

結論は結果により妥当な裏付けを得ていますか。

一部該当

研究設計は適切で学術的価値がありますか。

はい

Reviewer Expertise:

言語学、コミュニケーション論、日本語学

I confirm that I have read this submission and believe that I have an appropriate level of expertise to confirm that it is of an acceptable scientific standard.

# Peer review: Reviewer response for version 2

**DOI:** 10.5256/f1000research.134652.r142987

**Published:** 2022-07-11

**Authors:** Mieko Sawada

**Affiliations:** 1 Faculty of Arts and Sciences, Kyoto Institute of Technology, Kyoto, Japan

本論文が改訂されたものは、「対象集合についての詳細な議論」に踏み込んだものとなっており、特に「“遊離数量詞が表すのは「ばかり」が設定する（主観的な）集合に含まれる事物の数量である”こと」は非常に明確で妥当なものであると考える。

（該当する場合は要回答）統計分析および解釈は適切ですか。

はい

本研究は明確かつ正確に提示されたものであり、最新の文献を引用していますか。

はい

方法と分析について第三者による再現が可能となるよう十分な詳細が提示されていますか。

はい

結果の基礎となるソースデータはすべて入手可能で再現性を十全に保証していますか。

はい

結論は結果により妥当な裏付けを得ていますか。

一部該当

研究設計は適切で学術的価値がありますか。

はい

Reviewer Expertise:

認知言語学、日本語学

I confirm that I have read this submission and believe that I have an appropriate level of expertise to confirm that it is of an acceptable scientific standard.

# Peer review: Reviewer response for version 1

**DOI:** 10.5256/f1000research.59172.r112372

**Published:** 2022-01-21

**Authors:** Yasuto Kikuchi

**Affiliations:** 1 Faculty of Letters, Kokugakuin University, Tokyo, Japan

１　査読者による要約

 先行研究では、バカリは「限定」を表すとしばしば主張されてきたが、その一方で、バカリは「非該当例」を許容するとも指摘されており、この点で「限定」と捉えるのは不適当であるという指摘も行われてきた。この論文は、(1) 遊離数量詞を含む文のバカリは「非該当例」を許容しないという事実を指摘した、(2) (1)の事実について、なぜそうなのかという理由の説明を提示した、(3) (1)の事実をもとに、バカリは「限定」を表すと捉えるべきであると主張した［4.2］、の３点が骨子である。

 ２　評価できる点

 (1)の事実の指摘［2.3.後半］は、最も評価してよい点である。

 なおまた、取り上げるべき先行研究にほぼ粗漏はなく（ただし，後掲10参照）、先行研究への理解も適正のようである。

 ３　評価できるというほどでもないが、問題ではない点

 （2）の理由の説明［3.3］は、内容としては成立はしているように思われる。

 ただ、こうした趣旨のことを述べるのに、ここまで難しげに述べなければならないものか、という印象はある。多数の文法研究者に、こうした趣旨で説明文を書いてほしいという課題を課したら、もっとすっきりした答案がありうるように思う。その意味で、内容的には問題はないにせよ、評価できるというほどでもない、というレベルにとどまっている。

 ４　問題点

 上記(3)の主張［4.2］は、残念ながら、十分な説得力をもつ論証を伴っていない。この点について、著者に理解してもらうために、以下に詳述する。

 ５　問題点の詳述

 (1)により明らかになったのは、

 ア．遊離数量詞がない場合は「非該当例」が許容される（「限定」とはいえない）。

 イ．遊離数量詞がある場合は「非該当例」が許容されない（「限定」である）。

 ということである（アは以前から知られていて、今回この論文が明らかにしたのがイ）。

 これは、図式的にいえば、

 　　　ア．　Aの場合はP。

 　　　イ．　B（Non A）の場合はQ（nonP）。

 というケースである。

 事実はここまでであり、ここで、もし、ア・イのうちどちらか一方を「普通のケース」と見る、という見方を採らないのであれば、上記ア・イのまま併記して終わりにすることもできる。

 だが、ここで、仮にアとイのどちらかが一般的・本来的な在り方（普通のケース）で、他方が例外的な・変則的な在り方だと見ようとするならば、AとBを比べて、いわゆる無標（unmarked）な場合のほうを一般的・本来的な在り方と見、有標（marked）な場合のほうを例外的・変則的と見るのが、普通に採られている見方であろう。遊離数量詞を含む文と含まない文とを比べ、一方を無標、一方を有標とせよと言われたら、大抵の言語研究者は、遊離数量詞を含まないほうを無標、含む文を有標と見るはずである。だとすれば、この場合はアを無標と見る、すなわち、

 ［Ⅰ］遊離数量詞を含まない（＝普通の）ケースでは、バカリは非該当例を許容する（「限定」ではない）。

 　ただし、遊離数量詞を含む場合は、非該当例を許容しない（「限定」的な意になる）。

 という見方が順当である。これは、言語研究者の多くがごく普通に採る見方のように思われる。

 　　これを逆にして、

 ［Ⅱ］遊離数量詞を含む場合は、バカリは非該当例を許容しない（「限定」である）。

 　ただし、遊離数量詞を含まない場合は、非該当例を許容する（「限定」でなくなる）。

 という見方を採るとすれば、［Ⅰ］と［Ⅱ］は、どちらを但し書きにするか、つまり、どちらを普通と見るかが逆である、ということになる。

 バカリの基本義を「限定」と見るという主張は、［Ⅱ］の見方を採った場合にのみ行える主張である。著者が前掲(3)のように「バカリ」の基本義を「限定」と主張しているということは、つまり、著者は［Ⅱ］の見方を採っているのだ、と見られる。だが、上述のように普通は［Ⅰ］のように見るところなので、［Ⅰ］ではなく［Ⅱ］のように見るためには、相応の根拠が必要なはずであるが、この論文では、それが示されないまま［Ⅱ］が採られているように読めた。

 さらにいえば、著者は、

 「遊離数量詞を含む文のほうが、遊離数量詞を含まない文よりも、文として「普通」の文である」

 という主張をしている（しかも、根拠を示さずにそう主張している）のと、実は、同じことなのではないか、と査読者には見える。この点に大きな違和感を感じる。

 一方、著者は、上記要約で査読者が(2)としてあげたように、〈遊離数量詞を含む文で「非該当例」が許容されない理由〉を説明しているが、実は、これは、遊離数量詞を含まない文を「普通のケース」と見た上で、遊離数量詞を含む文ではそのように行かないことの理由を説明した、という発想のものなのではないか。つまり、著者自身、実は［Ⅰ］の見方に拠っているのではないか、とも思われる（だとしたら、「限定」を基本義と見るのは成り立たない）。もし、著者の基本的な主張のとおり［Ⅱ］の見方を採るなら、その場合は、〈遊離数量詞を含まない文で「非該当例」が許容される理由〉のほうを丁寧に説明すべきことになる。その説明が行われていない点も不備であろう。

 察するに、著者には、バカリを「限定」と見たいという潜在的な意識があり、それに好都合な遊離数量詞のケースを発見したので（ここまではいいのだが）、その遊離数量詞のケースをスタンダードのように捉えて残りの議論を展開し、望んでいた「結論」に到達させた、というケースなのではなかろうか。だが、後段の議論には、上述のように無理があり、成立していないと言わざるを得ない。上記ア・イのような事実が観察された場合、どちらが一般的・本来的な在り方かを合理的に見極めるべきところ、それを欠いたまま（厳しく言えば、冷静で合理的な「大局観」を欠いたまま）、いわば論点先取的に遊離数量詞を含むほうを本来的な在り方と見てしまった、というケースかと推測される。

 ６　承認ステータスについて

 　本プラットフォームの承認水準がどのぐらいのものなのか、初めての査読であるため判断できないところがあるが、一応、学内の紀要などではないレベル（学会誌並みのレベル）であるなら、残念ながら、以上のように論証に不十分な点があり、そのままの形での承認には至らない。むしろ、このまま承認し公開を続けることは、著者への評価を下げることになりそうに思われる（→次項）。

 　しかし、(1）の事実の指摘には意義があるので、不承認とはせず、条件付き承認としておく。相当根本的な修正が必要であり、その方向性を下記に助言しておく。

 ７　改善の方向など

 論証に問題がある論文については、普通は論証をしっかり補強するようにという助言を行うところであるが、この論文では、「限定が基本義である」とする議論を成立させることは困難である、と査読者は見る。

 そこで、冒頭の要約に示した(1)(2)(3)のうち、(3)は取り下げるべきだと考える。(1)を指摘し、（2）でその理由を説明するだけでも、地味ではあるが、十分立派な論文である。確実に正しいところまでで止めるというのは、研究者として重要な姿勢である。(3)を主張した途端に、少なからぬ読者に、怪しげな論、あるいは強弁だという印象を与えてしまい、著者への評価を下げることになりかねないのではないかと、査読者はその点をおそれるものである。

 査読者は、この論文は、次のように仕立て直すのがよいのではないかと考える。：

 ・(1)を指摘する。

 →・「そうはいっても、遊離数量詞がない場合が、基本義が反映されている場合だと見るべきである。」と確認する。（先にア・イなどとして説明した箇所を参考にされたい）

 →・「では、遊離数量詞がある場合にはなぜ……?」という問を立てて、（2）に相当する議論を展開する。投稿者の(2)の議論は上で批評したようにやや難解であるが、要するに、「バカリは、基本的には〈認識的な際立ち〉に基づいて〈数多く観察される〉ことを表すだけなので、非該当例を許容しうるが、数量詞を伴った場合は、数量情報との合致が求められるため、正確な限定を表す結果となる」という趣旨の説明をすればよいと考える。

 →・その上で、遊離数量詞がある場合に「限定」的になることについては、このように説明がつくのだから、バカリの基本義を「限定」でないと見ること自体は問題ない（むしろ支持される）、というふうにまとめる。

 これが穏当な道だと査読者は考える（あくまでも査読者の意見であるが）。

 ８　論の補強のための参考

 「先行研究では……「ばかり」がそれ（＝非該当例）を許容しない環境があることについて指摘・考察した研究は管見の限り存在しない。」［2節末］とあるが、実は、否定を伴う場合には、「ばかりでなく」は「だけでなく」と事実上同義である、ということは、多くの日本語教授者が承知していて学習者にも教えていることである。研究としての明記はないかもしれないが、これは、「非該当例の許容」のない意味でのバカリを否定している（だから「該当例が他にもある」という意味になる）と見るべきケースであり、こういう難しい言い方はしていなくても、事実としては知られてきたと言いうるものである。

 数量詞と否定が「お友達」である、というふうに見える言語現象は、あれこれあるかと思われ、あわせて検討してはどうか。

 なお、数量詞や否定絡みで不思議なことが起こる場合（例えば、生成文法の古典期に「変形は意味を変えない」と言っていたときに、manyやsomeが絡む受身文では、能動文とは違う意味になる（スコープ絡み）、ということが指摘された）は、あくまでも、その変わったことが起こる場合が有標なケースである、という理解をすべきことは、念のため付言しておく。

 ９　その他の問題点：

 ■　例文の適否判断に疑問がある点［2節末］：

 (17) 【女性を 400 人，男性を 100 人招待した場合】

 a. # 女性 ばかり 500 人招待した。

 b. 招待した 500 人は女性 ばかりだ。

 査読者の語感ではbも#である。［だとすると、この論文の主張（この査読の冒頭で示した要約中の(1)）は、「遊離数量詞」だけについてではなく、「数量詞」全般について成り立つことになる。］　このようなデリケートなケースでは、アンケートの実施が必要であろう。

 なお、冒頭に査読者が（２）としてあげた理由の説明について、著者は「事態」ということに依存した説明を考えているが、上記７の中で述べたように、「数量情報との合致が求められる」ということがポイントなのではないかと思われ、「事態」への依存は必ずしも必要ではないように思われる。だとすれば、遊離数量詞でない数量詞について同様の現象が観察されても困らないのではないかと思われ、事実に誠実な論を組み立てることを薦めたい。

 ■　日本語記述文法研究会編 (2009) の紹介と論評の部分［4.1.1］

 　著者のこの箇所の骨子に問題はないが、要するに、「同書は、(30)を(33a)と(33b)に分けられるかのように見せているが、この部分を一読するだけで、（33b）が(30)の下位区分になっていないことは、すぐ見て取れる。」というケースなので、もっと簡単に（失礼にならない程度に）、そのような趣旨のことを述べてあっさり片づけてよいのではないか。「注目に値する」と持ち上げるほどのものではないと思う（気を遣ったのかもしれないが、厳然と否定すべきケースだと思う。

 なお、(32)の下、「理論的矛盾」とあるのは「論理的矛盾」では ?

 ■　澤田 (2007) の紹介と論評の部分［4.1.2］

 　澤田の指摘（の、少なくとも方向）は、ごく全うなものだと査読者には評価できる。

 ただ、遊離数量詞の文のバカリについては、その時点で留意されていなかったので、それにかかる補いが必要である、というだけのことであり、この小節のタイトルを「澤田（2007）の指摘とその問題点」とするのは、多少の違和感がある。小さなことではあるが、こう書くと、澤田（2007）の立論自体にすでに不備を抱えていたかのような印象を受けてしまう。遊離数量詞についてはこれまでの先行研究は誰も気づいていなかったのだから、それはやむを得ないであろう。これをカバーできていなかったことを問題点というなら（確かに問題点ともいえるが）、全ての先行研究について「○○（xxxx）の問題点」と言わなければならないことになる。

 　この節は、遊離数量詞の問題に出会う前から、「限定（のように見える場合があること）」と「非該当例があること」を、どうやって料理するかという問題に取り組んできた研究があるのだ、という話を中核にし（4.1節のタイトルもそのようなものにするのがいいように思う）、日本語記述文法研究会編 (2009) と澤田（2007）を紹介すればよく、両者ともに遊離数量詞については扱っていないということについては、ここでは過大に問題視しなくていいように思う。

 また、4, 2の冒頭に、この両研究について「いずれにおいても問題がある」という整理になっているが、澤田は（遊離数量詞の問題に気づいていない点を別にすれば）相応に料理できていたのに対し、日本語記述文法研究会のほうは成功していないので、「問題」とはいっても、だいぶ水準が違うのではないか。このまとめ方はいかがかと思う。

 なお、澤田の紹介にあたり、著者は「澤田は……限定(的解釈)はそこから「派生」する「二次的な効果」であると捉えている。つまり、限定は「ばかり」の意味ではなく、言わば語用論的効果であるとしているのである。」と述べているが、細かい点ながら、「派生」「二次的」ということをあっさり「語用論的」と言い換えてよいのかは疑問である。「派生」「二次的」ということは、「意味論」の世界の中でも起こりうることであり、本件に関しては、そう捉えるだけでも、議論が成り立つのではないかと思われる。澤田が、この「派生」「二次的」を意味の問題と考えているのか、語用論の問題と考えているのか、あるいはその点は不問に付しているのかは、わからないというべきであろう。「語用論的」と（勝手に）言い換えることには慎重でありたい。

 10　参考文献

 以下を加えてもよいのではないか。

 安部 朋世「バカリによる「限定」」『和光大学表現学部紀要』 2000，1，pp.135-144，和光大学表現学部．

 以上

（該当する場合は要回答）統計分析および解釈は適切ですか。

対象外（統計を使っていない）

本研究は明確かつ正確に提示されたものであり、最新の文献を引用していますか。

はい

方法と分析について第三者による再現が可能となるよう十分な詳細が提示されていますか。

はい

結果の基礎となるソースデータはすべて入手可能で再現性を十全に保証していますか。

ソースデータは不要

結論は結果により妥当な裏付けを得ていますか。

一部該当

研究設計は適切で学術的価値がありますか。

一部該当

Reviewer Expertise:

日本語学、特に日本語文法。日本語教育。

I confirm that I have read this submission and believe that I have an appropriate level of expertise to confirm that it is of an acceptable scientific standard, however I have significant reservations, as outlined above.

# Response

**Published:** 2022-05-31

**Authors:** YOSHIKO NUMATA

**Affiliations:** University of Tsukuba, Japan

菊地先生

 お忙しい中，拙稿の査読のために貴重なお時間を割いていただき，誠にありがとうございました。早速多くのご意見を頂いたにもかかわらずご回答が遅くなってしまい，大変失礼いたしました。以下，頂いたご意見につきまして，可能な限りご回答申し上げます（以下ではご意見を頂いた原稿を「第1稿」，修正した原稿を「第2稿」と呼称しております）。なお，すべてのご意見に対して十分なご回答をご用意することはできませんでしたが，第2稿では構成・論じ方を含めて修正を施しました。依然として不十分な点があるかと存じますが，その点につきましては改めてご指摘いただければ幸いです。

1．「5 問題点の詳述」におけるご意見について

 ご意見をくださりありがとうございます。確かに，第1稿では，「ばかり」は遊離数量詞と共起する場合に「限定」を表し，遊離数量詞と共起しない場合は「限定」を表さないかのように述べられております。しかし，これは筆者の述べ方の不備によるものであり，本来の意図はそうではありませんでした。

 筆者は，「ばかり」が遊離数量詞と共起する場合に非該当例が許容されないという現象について，“「ばかり」は（遊離数量詞との共起の有無を問わず）集合の内部に非該当例が無いことを表す（＝「限定」を表す）”ということを示唆していると考えております。つまり，「ばかり」と数量詞の共起については，「ばかり」の「無標」のケース，あるいは「普通」のケースと捉えているのではなく，「ばかり」の意味について示唆的な現象を観察することできるケース（の1つ）と捉えております。第2稿では述べ方を全面的に修正し，この点が明確になるようにいたしました。

2．「9 その他の問題点　例文の適否判断に疑問がある点」におけるご意見について

 確かに，第1稿（17b）を（17a）と同様に「#」と判定する話者が存在する可能性は否定できません。そうであれば，ご指摘いただいたように「数量情報との合致」が重要ということになると考えられます。一方で，相対的にはやはり（17b）の方が文脈的自然度は高いとも考えております（第2稿注12）。つまり，「数量情報との合致」が重要ではあるものの，その情報が「事態」の数量である場合にはより強固な「合致」が求められると考えております。

3．「9 その他の問題点　澤田（2007）の紹介と論評の部分」におけるご意見について

 確かに，澤田（2007）は「ばかり」の振る舞いについて遊離数量詞と関連付けて議論しているわけではなく，それはご指摘の通り「その時点で留意されていなかった」ためであると思われます。しかし，澤田（2007）について，第1稿では遊離数量詞が生起する場合の現象に触れていない研究として取り上げているわけではなく，“「ばかり」の意味は「限定」ではないと主張する研究”として取り上げております。これは第1稿の主張と対立するものであるため，「澤田（2017）の指摘とその問題点」というタイトルで取り上げた次第です。

 ただし，第1稿は筆者の意図が十分に伝わりにくい記述になっておりました。また，第1稿における澤田（2007）の位置づけには不十分な点がございました。これらを踏まえ，第2稿では澤田（2007）との関係性に関わる部分の記述を修正いたしました（第2稿4.2節・4.3節）。

 なお，ご指摘の通り，澤田（2007）による「派生」「二次的」という表現を「語用論的」と言い換えていたことは不適切でしたので，第2稿ではこれを削除いたしました（第2稿4.2節（35）の直後）。ご指摘いただきありがとうございました。

4．「10 参考文献」におけるご意見について

 安部（2000）を参考文献に加えました（第2稿注17，参考文献欄）。ご指摘いただきありがとうございました。

# Peer review: Reviewer response for version 1

**DOI:** 10.5256/f1000research.59172.r112374

**Published:** 2022-01-06

**Authors:** Mieko Sawada

**Affiliations:** 1 Faculty of Arts and Sciences, Kyoto Institute of Technology, Kyoto, Japan

本論文は、「ばかり」が非該当例を許容しない現象があることを指摘した点は、学術的新規性が高いと判断する。本論文が主張するように、（27）は非該当例を含まない例として解釈できる。

 （27） 白いシャツばかりを1000 枚購入した。

 しかしながら、「ばかり」が遊離数量詞と共起する場合でも非該当例を含む場合がある。　

 例えば、Aの例は釣り好きの友人の発話である。

 A：この間、カワハギばかりを10枚釣ったよ。

 Aの発話は、「ばかり」が遊離数量詞と共起している例である。筆者がAの発話に対して、「カワハギ以外は釣れなかったのか」と尋ねたところ、「外道（本命以外の魚）も何匹か釣った」という回答だった。つまり、遊離数量詞の場合でも非該当例を含む場合があるということである。

 Bはガチャに凝っている友人に聞いたところ、Ｂの発話は自然であるということであった。

 B：10回ガチャやったら、サルばかりが5匹出た。

 Bの発話も遊離数量詞の例である。Bは10回ガチャをやって、サルが5回でた場合の発話であった。釣り好きの友人もガチャに凝っている友人も日本語の母語話者である。

 　では、（27）とA、Bの例の違いについて考えてみたい。（27）を他者の発話として聞き、非該当例を含まないと解釈する場合は、他者が1000枚の白いシャツを買ったと解釈した場合である。また（27）が話し手の自伝的記憶を想起して発話された場合は、話し手は事態をコントロールしており、意図的に1000枚購入している。一方、AとBの共通点は、いずれの話し手も自伝的記憶を想起しており、釣りもガチャも話し手のコントロールが及ばないことを認知しており、複数回行っているという点である。

 　この現象は、「ばかり」が遊離数量詞と共起する場合の発話でも、話し手が自伝的記憶を想起して、行為が複数回であった場合、非該当例を含む場合があることを示唆している。このように、「ばかり」を使用した文が非該当例を含まない解釈は特別な条件が必要であり、 「ばかり」の意味は非該当例を許容しない「限定」と考えるのが妥当であるという主張が理解できない。ゆえに、「ばかり」の意味を「限定」と位置づけることの意義を示してほしい。筆者は、日本語の認知言語学の発展のためにも、「ばかり」のように意味の分化が非常に興味深い不変化詞は、様々な観点から研究をしていくことが有意味であると考える。ゆえに本論文で、今後の方向性と示されている「対象集合についての詳細な議論」は非常に興味深いと考える。

（該当する場合は要回答）統計分析および解釈は適切ですか。

はい

本研究は明確かつ正確に提示されたものであり、最新の文献を引用していますか。

はい

方法と分析について第三者による再現が可能となるよう十分な詳細が提示されていますか。

はい

結果の基礎となるソースデータはすべて入手可能で再現性を十全に保証していますか。

はい

結論は結果により妥当な裏付けを得ていますか。

一部該当

研究設計は適切で学術的価値がありますか。

はい

Reviewer Expertise:

認知言語学、日本語学

I confirm that I have read this submission and believe that I have an appropriate level of expertise to confirm that it is of an acceptable scientific standard, however I have significant reservations, as outlined above.

# Response

**Published:** 2022-05-31

**Authors:** YOSHIKO NUMATA

**Affiliations:** University of Tsukuba, Japan

澤田先生

 お忙しい中，拙稿の査読のために貴重なお時間を割いていただき，誠にありがとうございました。早速多くのご意見を頂いたにもかかわらずご回答が遅くなってしまい，大変失礼いたしました。以下，頂いたご意見につきまして，ご回答申し上げます。なお，以下ではご意見を頂いた原稿を「第1稿」，修正した原稿を「第2稿」と呼称しております。

「ばかり」が遊離数量詞と共起しても非該当例を含む場合があるというご指摘について

 確かに，ご提示いただいた①②の例はサル以外のキャラクター（非該当例）が出た場合やカワハギ以外（非該当例）が釣れた場合でも成立すると思われます。

 ①    10回ガチャやったら，サルばかりが5匹出た。

 ②    この間，カワハギばかりを10枚釣ったよ。

 しかし，“「ばかり」が遊離数量詞と共起した場合は非該当例を許容しない”という筆者の主張は，①の例で言えば，10回出たキャラクターの中にサル以外（非該当例）は含まれないということではなく，“遊離数量詞「5匹」が表す数量の中にサル以外（非該当例）は含まれない”ということを示すものです。同様に，②の例で言えば，“遊離数量詞「10枚」が表す数量の中にカワハギ以外（非該当例）は含まれない”ということを示すものです。特に①の例は「サルが5回でた場合の発話」ということでご紹介いただきましたので，これは筆者の主張を支持する例であると考えております。しかし，第1稿では筆者の主張がやや曖昧になっている箇所がございましたので，第2稿ではその点を修正いたしました（第2稿1節（5）の直後や2.3節（19）などを始めとする複数箇所）。

 また，確かに①②の例と次の③の例は「話し手のコントロール」や行為の複数性において差が認められると言えます。

 ③    白いシャツばかりを1000 枚購入した。

 しかし，前述の通り，筆者の主張は遊離数量詞が示す数量の中に非該当例が含まれないというものであり，その点では①②と③の間に差は認められません。従って，少なくとも「話し手のコントロール」が及ぶ事態であることと「行為が複数回」でないことを指して「特別な条件」とする場合においては，「『ばかり』を使用した文が非該当例を含まない解釈は特別な条件が必要」ということにはならないと考えます。

 一方，ご指摘いただいた内容は，遊離数量詞が表す数量と事態の総数量との関係において，①②の例と③の例で差があるということを示していると理解いたしました。具体的には，①②の場合は「5匹」「10枚」が「出た」「釣った」の総数量と（必ずしも）一致しない（出た数＞5匹，釣った数≧10枚）のに対し，③の場合は「1000枚」が「購入した」の総数量と基本的に一致する（購入した数＝1000枚）ということです。つまり，ご指摘に照らせば，「話し手のコントロール」が及ばない事態，かつ「行為が複数回」である場合（①②）は遊離数量詞が表す数量と事態の総数量が（必ずしも）一致せず，反対に「話し手のコントロール」が及ぶ事態，かつ「行為が複数回」でない場合（③）はそれらが基本的に一致するということになります。これは数量詞研究で言われるところの「全体量」と「部分量」の議論にも関わる可能性があり，大変興味深い現象ですが，今回の議論の範囲を超えていると判断し，第2稿では扱いませんでした。

# Peer review: Reviewer response for version 1

**DOI:** 10.5256/f1000research.59172.r112370

**Published:** 2022-01-06

**Authors:** Takuzo Sato

**Affiliations:** 1 Gakushuin Women's College, Tokyo, Japan

「ばかり」の意味解釈に数量詞をからませて分析している着眼に新規性があり、非常に興味深い。また、「ばかり」の、機能をあくまで「限定」として位置づけたうえで、例外的ともみえる現象に対して統一的な説明を与えようとする結論も説得的である。

 唯一、問題点として残るのは、鍵となる例文の解釈が恣意的で説得力に欠ける点である。数量詞の働きにより非該当例解釈が許容されない例文の解釈には再考の必要性があるのではないだろうか。

 例えば、例文（23）の「100枚のシャツを購入した」に関して、（23’）において「計1000の「購入する」という事態が生じた」としている。しかしながら、（23）のデフォルト解釈はむしろ「「1000枚のシャツ購入」という事態が1回生じた」ではないか。（23）において非該当例解釈ができないのは、先行研究のいう「複数制の制約」によるとみるのがより自然である。

 このような疑問が生じないような説明を与えるか、もしくは上述の線から数量詞による非該当例解釈の阻止という事実に光を与えるべきと思われる。

（該当する場合は要回答）統計分析および解釈は適切ですか。

一部該当

本研究は明確かつ正確に提示されたものであり、最新の文献を引用していますか。

はい

方法と分析について第三者による再現が可能となるよう十分な詳細が提示されていますか。

はい

結果の基礎となるソースデータはすべて入手可能で再現性を十全に保証していますか。

はい

結論は結果により妥当な裏付けを得ていますか。

はい

研究設計は適切で学術的価値がありますか。

はい

Reviewer Expertise:

現代日本語の文法論および意味論

I confirm that I have read this submission and believe that I have an appropriate level of expertise to confirm that it is of an acceptable scientific standard, however I have significant reservations, as outlined above.

# Response

**Published:** 2022-05-31

**Authors:** YOSHIKO NUMATA

**Affiliations:** University of Tsukuba, Japan

佐藤先生

 お忙しい中，拙稿の査読のために貴重なお時間を割いていただき，誠にありがとうございました。早速多くのご意見を頂いたにもかかわらずご回答が遅くなってしまい，大変失礼いたしました。以下，頂いたご意見につきまして，ご回答申し上げます。なお，以下ではご意見を頂いた原稿を「第1稿」，修正した原稿を「第2稿」と呼称しております。

1．非該当例解釈が許容されない例文の解釈について

 ご指摘の通り，「シャツばかり1000枚購入した」の一般的な解釈は「“1000枚のシャツ購入”という事態が1回生じた」であると思われます。それにもかかわらず「“購入”という事態が1000回生じた」という解釈を示したのは，いわゆる「探索」が1000回行われた（遊離数量詞の示す数が「探索」の数と一致する）ということを示す意図がございました。

 なお，筆者はこのように「探索」の数（「ばかり」が形成する主観的集合の要素の数）に関与するのは遊離数量詞（副詞位置に生起する数量詞）に限られると考えておりました。例えば，女性400人と男性100人を招待した場面では，次のように遊離数量詞が生起する①は不自然であり，そうでない数量詞（以下，非遊離数量詞）が生起する②は自然であると考えておりました。

 【女性を400人，男性を100人招待した場合】

 ① # 女性ばかり 500 人招待した。

 ②    招待した 500 人は女性ばかりだ。

 しかし，菊地先生に頂いたご意見により，当該の場面では②も不自然と判定する話者も存在することが分かりました。これは，非遊離数量詞も「探索」の数に関与し得ることを示唆しております。

 ただし，そうした話者が存在しても，少なくとも①よりは②の方が許容されやすいと考えております。つまり，“数量詞が生起した場合は，それが示す数が「探索」の数と一致し得るが，特に遊離数量詞は（「探索」と親和性がある「事態」と密接に関わるため）その含意が生じやすい”と考えております。

2．非該当例許容解釈の不成立と複数性の制約について

 筆者は「複数性の制約」について，概ね“「ばかり」は事態（≒探索）が複数である場合にのみ用いられる”という制約であると理解しております。「シャツばかり1000枚購入した」で言えば，例え1000枚のシャツを一度に購入した（行為の回数＝単数）という場合でも，「シャツである」という結果が得られる「探索」が“複数”行われていれば当該の文が成立するため，その意味では今回の考察課題にも「複数性の制約」が関わっていると捉えられます。

 しかし，非該当例許容解釈ができない要因と「複数性の制約」については関係性を見出しておりません。筆者の理解が及んでいない，あるいはそもそも当該の制約に関する理解が不十分であるという可能性がありますが，少なくとも現時点ではそのように考えております。大変恐れ入りますが，修正した第2稿を今一度ご確認いただき，非該当例許容解釈ができない要因についてはやはり「複数性の制約」と関連づけて説明する方が自然という場合には，改めてご指摘いただきたく思います。

# Peer review: Reviewer response for version 1

**DOI:** 10.5256/f1000research.59172.r112371

**Published:** 2021-12-20

**Authors:** Toshiyuki Sadanobu

**Affiliations:** 1 Faculty of Letters/Graduate School of Letters, Kyoto University, Kyoto, Japan

論文を読ませていただきました。遊離数量詞構文において「ばかり」が「非該当例」を許容しなくなるという観察は意義あるものと判断します。が、この論文が（賛否は別として）一つの論考として成り立つには、クリアしなければならない問題もあると判断し、「条件付き承認」と判定します。以下、問題について説明し、提案を書きます。ご参考になれば幸いです。

 問題１：前提とされている概念「意味」がはっきりしない。

 論文では、「「ばかり」の「意味」とは何か？」という論点が設定され、この論点をめぐる形で考察が展開されています。が、その「意味」とは、どういうものを含み、どういうものを含まないのでしょうか？　ある説を対立説（要旨のことばで言えば「「ばかり」の意味を限定ではないと示唆・主張する研究」）と位置付けて反駁したり、自説を主張したりするには、まずこの点が明らかにされる必要があると考えます。

 仮にある研究者が「「ばかり」は限定を意味する」あるいは「「ばかり」は限定を意味しない」と明記していても、その研究者の「意味」の概念を、著者の「意味」観と比べ、対応づけを検討しなければ、その研究者の見解を自説の仲間、あるいは対立説に位置付けることはできないでしょう。

 論文が挙げている先行研究の中で、対立説と位置付けられているものは唯一、澤田(2007)だけですが、これも、本当に対立説と言えるのか、論文を読んでいて確信できませんでした。というのは、澤田(2007)の考えとして引用されている第4.1.2節の(35)は、「ばかり」を発する話し手の動機（「目的」）について述べられたものであって、「ばかり」の意味について述べられたものではないからです。その末尾の部分には、限定的解釈が「派生的」にせよ「でてくる」とも書かれています。これを本当に、「「ばかり」は限定を意味しない」と述べたものと読み込んでよいのでしょうか。

 以上のことを、著者自身自覚されているのではないかと思わせる、記述の弱さが論文には見られます。もし、どうしても澤田(2007)を対立説とみなして「ばかり」の意味論にこだわるのであれば、それらは改めるべきでしょう。具体的に言うと、要旨欄の「示唆・主張」は「示唆・」を削除して「主張」とするべきでしょうし、対立説は第4.1.2節ではなく第1節で真っ先に紹介すべきでしょう。第2節末尾の「ばかりの意味についても考察する」は、これこそがメインのはずですから、「も」は削除すべきでしょう。

 問題２：遊離数量詞を持ち出す意義がはっきりしない。

 遊離数量詞を持ち出す意義も、はっきり理解できませんでした。これも、論点として「ばかり」の意味論が設定されている結果ではないでしょうか。というのは、「「ばかり」の意味＝限定」説は、遊離数量詞を持ち出さなくても、他の、ずっと簡単な形でも主張できるからです。以下、それを具体的に２つ述べます。

 その１：「厳密に」「厳密な話」などの語句が「～ばかり」にかかるだけで、「非該当例」は許容されにくくなります。例：うどん以外も食べていた場合、「先週はうどんばかり食べてたよ」と比べて「先週は厳密な話、うどんばかり食べてたよ」は自然さが低い。

 その２：「非該当例」の存在が会話相手に知られた場合、相手に反駁され得ます。例：うどん以外も食べていたことが聞き手に知られた場合、「先週はうどんばかり食べてたよ」と言えば、「うどんばかりじゃないじゃん。×××も食べてたじゃん」などと反駁される可能性があります。

 いずれも、「非該当例」が、いわば「非公式のもの」でしかないことを示すものです。こうした「非該当例」の「非公式性」は、多くの研究者に共有されており、（「意味」の定義はさまざまであれ）「「ばかり」の意味＝限定」説は広く受け入れられているものではないか、というのが評者の認識です。

 問題３：仮説がアドホックに感じられる。

 評者の理解によれば、著者は、遊離数量詞の効果で「非該当例」が許容されなくなるという自身の観察を、次の２段階の仮説によって説明することで「ばかり」の意味論につなげようとしています。以下、評者のことばで述べます。（ちなみに「「現実世界の事態の数量」との（不）一致」という表現には、改善の必要を感じます。「もしあの時、白いシャツばかり10枚買っていたなら～」のような、仮定世界や反事実世界の話をも「現実世界」と言わねばならないことになってしまうからです。）

 段階１：「ばかり」の話し手が想定する集合は、２種類あり得る。集合は、「当該文脈で想定される候補の集合」（＝とりたて表現一般に想定される集合で、「非該当例」を含む）とは別に、「非該当例を排除した集合」でもよい。

 段階２：「ばかり」の文に遊離数量詞が現れ、事態の数が明示される場合は、「非該当例を排除した集合」が想定できず、「当該文脈で想定される候補の集合」しか想定できなくなる。にもかかわらず、この場合、「非該当例」は許容されない。（だから「ばかり」は限定を意味する。）

 ここでは、以上の２段階のうち、段階１について述べます。

 この仮説（段階１）は、「限定を意味するはずの「ばかり」が「非該当例」を許容する」という謎を、「「ばかり」の話し手が想定する集合としては、「非該当例」を排除した集合が許容される」という、別の謎に変換しています。この変換については、佐藤(2017)が参考にされているとはいえ、論拠が出されておらず、アドホックに感じられます。

 提案：諸説の統合

 以上の３つの問題を回避あるいは解決し、この論文を一つの論考として成り立たせるための提案をおこないます。（単なる提案ですので、却下していただいても構いません。）

 それは、「「ばかり」の意味とは限定か、そうでないのか？」という論点の代わりに、「「ばかり」の非該当例が許容される場合と許容されない場合の違いとは？」という別の問題を立て、この問題に、これまでの諸説を統合する形で解答を与える、ということです。

 問題３について、論文では、仮説(段階１)の論拠が出されていませんが、これに強く関連する概念「集合」については、菊地(1983)に始まる先行研究の言及があります。これを利用すればどうでしょうか。評者のことばで言えば、それは（(8)に書いていただいているように）「探索の集合」ということです。探索とは体験の中核を占めるもので、探索の集合を語るということは、結局、体験の集合を語るということです。体験談では、その体験を「語るに足る」(つまりreportableな）ものにするために、嘘と思われない程度の誇張や脚色がなされやすいと考えると(Labov 2001)、ある偏り（例：先週の自分の食生活の偏り）を表すのに、知識として語らず、その知識を探る個人的な体験として語る場合、非該当例が許容されやすいということも、自然なこととして理解できるのではないでしょうか。

 なお、探索と体験については、たとえば拙著(定延2016)をご覧いただければと思います。そこでは、ある料理について「からいばかりで少しもおいしくない」と言う場合のような、形容詞＋「ばかり」についても取り扱っています。

 要望：非該当例が許容されなくなる根本原因について、さらに論じていただきたい。

 上記の段階２について述べます。著者は、遊離数量詞で事態の数が明示されると、「非該当例を排除した集合」が想定できなくなる、と論じています。が、事態の数が明示されると、なぜそのような効果が得られるのでしょうか。つまり、事態の数として、非該当例を含んだ数がなぜ表示できないのでしょうか？

 論文はこの問題について論じておらず、そのため、現象を説明しているのか、説明すべき現象を単に別の形で言い換えているに過ぎないのか、はっきりしないというのが、率直な感想です。この問題について、論じていただきたい、その際、以下２点についても触れていただければというのが、評者の要望です。

 その１：上に記したように、「非該当例を排除した集合」を想定不能にするものとしては、いろいろなものがあり得ます。たとえば、「厳密な話」などの語句を挿入することです。またたとえば、相手に反駁され得ないような客観的な描写を話し手が心がけるだけでも、「非該当例を排除した集合」は想定されなくなります。遊離数量詞による事態の数の明示は、「非該当例を排除した集合」を想定不能にする根本的な原因ではなく、さまざまな要因の中の一つとして位置づけられるべきではないでしょうか？

 その２：もう少し言えば、遊離数量詞による事態の数の明示が「非該当例を排除した集合」を想定不能にするというのは、傾向であって、例外もある、と考えられないでしょうか？　著者は、「遊離数量詞」として、数詞から成るものばかりを挙げていますが、遊離数量詞としては、数詞の現れない、より感覚的なものも考えられます。以下の実例をご覧ください。

 まあね、中にはおっさんもいますけど、そうですねぇ、８割ぐらいが若いきれいな女の子なんですわ。もう、絶対顔で客選んでるやろ！っていうぐらい、きれいな子ばっかりの店なんですね。

 ［三好康之・ITのプロ46　2020『情報処理教科書 高度試験午後Ⅱ論述 春期・終期 第2版』 p. 63，翔泳社］

 この例で述べている状況は、「この店には、もう、絶対顔で客選んでるやろ！っていうぐらい、きれいな子ばっかりがいる」という文で表される状況で、この文には「もう、絶対顔で客選んでるやろ！っていうぐらい」という数量詞（と言って悪ければ数量詞句）が現れています。が、著者によればその割合は10割ではなく「８割」で、２割は「おっさん」などのようです。この例は別としても、たとえば「朝からおかしな答案ばっかり、山ほど採点してこっちまでおかしくなる」などと言うことは、「山ほど」採点した答案の中にまともな答案が少しぐらい入っていても自然かもしれません。もしも仮に、遊離数量詞の中に、数詞から成るものと、そうではない、より感覚的なものの違いが見られるのであれば、それもまた、「ばかり」の体験性と関連していると考えられはしないでしょうか。

 見知らぬ異国の街をバスで走行中、車外の風景を眺めている観光客が、「この街にはあちこちにレストランがあるね」という意味で同乗者に「この街にはしょっちゅう、レストランがあるね」と言うのは、「この街には30秒に１軒、レストランがあるね」などと言うより自然だとすると（定延 2016）、そうした遊離数量詞の区別も、これと並行しているのかもしれません。このような可能性もご一考いただければ、ご論考がさらに内容豊かなものになると考えました。

 以上、勝手なことを書きましたが、理解の不足や誤りがありましたら失礼します。少しでもご参考になる部分があれば幸いです。

 Labov, William. 2001. “Uncovering the event structure of narrative.” In Deborah Tannen and James E. Alatis (eds.), *Georgetown University Round Table on Languages and Linguistics 2001*, pp. 63-83, Washington, DC: Georgetown University Press.

 定延利之　2016　『煩悩の文法―体験を語りたがる人びとの欲望が日本語の文法システムをゆさぶる話（増補版）』東京：凡人社

（該当する場合は要回答）統計分析および解釈は適切ですか。

対象外（統計を使っていない）

本研究は明確かつ正確に提示されたものであり、最新の文献を引用していますか。

はい

方法と分析について第三者による再現が可能となるよう十分な詳細が提示されていますか。

はい

結果の基礎となるソースデータはすべて入手可能で再現性を十全に保証していますか。

はい

結論は結果により妥当な裏付けを得ていますか。

一部該当

研究設計は適切で学術的価値がありますか。

はい

Reviewer Expertise:

言語学、コミュニケーション論、日本語学

I confirm that I have read this submission and believe that I have an appropriate level of expertise to confirm that it is of an acceptable scientific standard, however I have significant reservations, as outlined above.

# Response

**Published:** 2022-05-31

**Authors:** YOSHIKO NUMATA

**Affiliations:** University of Tsukuba, Japan

定延先生

 お忙しい中，拙稿の査読のために貴重なお時間を割いていただき，誠にありがとうございました。早速多くのご意見を頂いたにもかかわらずご回答が遅くなってしまい，大変失礼いたしました。以下，頂いたご意見につきまして，ご回答申し上げます。なお，以下ではご意見を頂いた原稿を「第1稿」，修正した原稿を「第2稿」と呼称しております。

1．「意味」と澤田（2007）について

 ご指摘の通り，第1稿では「意味」という概念が「どういうものを含み、どういうものを含まない」のかということを明示しておりませんでした。このご指摘は，第1稿において，特に澤田（2007）との差を“「ばかり」の意味を限定とするか否か”という点に求めていることと密接に関係すると思われます。ご指摘いただき誠にありがとうございました。ご指摘については，「意味」一般について十全な定義に及ぶことはできませんでしたが，澤田（2007）と本稿との違いを明確に示すように改稿しました（第2稿4.3節第3段落）。

 改めた記述の内容をまとめると次のようになります。澤田（2007）はとりたてる要素が「多い」（あるいは「多すぎる」）ということを伝えるのが「ばかり」にとって重要であり，「限定」はその「二次的な効果」と述べております。この記述につきまして，当該要素が「多い」場合に「ばかり」が用いられるという点は筆者の立場と一致しております。しかしながら，「多い」ということを伝えるのが「重要」であり，「限定」が「二次的」という位置づけについては筆者の立場と異なります。具体的に言えば，筆者は「限定」が「二次的」とは考えず，また，「多い」というのは「ばかり」が集合を形成するに当たっての前提条件であると考えております。

2．要旨欄と第2節の文言について

 ご指摘を踏まえ，第2節末尾の「ばかりの意味についても考察する」の「も」は削除いたしました（第2稿2.3節最終文）。なお，要旨を全面的に修正した都合上，第1稿の「示唆・主張」はそれ自体を削除いたしました。

3．遊離数量詞を持ち出す意義について

 筆者は“「ばかり」は主観的集合の内部に非該当例が存在しないことを表す”ということが遊離数量詞共起下の現象から明らかになると考えており，その点に遊離数量詞を持ち出す意義を見出しております。ご指摘を踏まえ，第2稿ではこの点が（少なくとも第1稿に比べて）明確になるように修正いたしました（第2稿2.3節（17）の直後）。

 ところで，ご教示いただいた2つの例のうち，特に「『非該当例』の存在が会話相手に知られた場合、相手に反駁され得」るという例は，ご指摘の通り「『非該当例』が、いわば『非公式のもの』でしかないことを示すもの」であると考えます（同様の例は佐藤（2017: 3-4）でも指摘されていることを確認しております）。しかし，この例では“「非該当例」は「ばかり」によって設定される集合の内部に存在するのか外部に存在するのか”という点までは十分に捉えられないものと思われます。これに対し，筆者は遊離数量詞共起下の現象を観察することで“「非該当例」は「ばかり」によって設定される集合の外部に存在する（内部には存在しない）”ということが明らかになると考えております。

 なお，「『厳密に』『厳密な話』などの語句が『～ばかり』にかかるだけで、『非該当例』は許容されにくくな」るというご指摘については検討の余地があると考えます。「厳密に」が介入すれば非該当例を許容しないということであれば，次の例文①②③はいずれも自然に成立することが予測されますが，「ばかり」と共起する③はやや不自然になるように思われます。

 ①    先週はうどんをよく食べてました。いや， 厳密にはうどん だけ食べてました。

 ②    先週はうどんをよく食べてました。いや， 厳密にはうどん しか食べませんでした。

 ③ ? 先週はうどんをよく食べてました。いや， 厳密にはうどん ばかり食べてました。

 これは，「ばかり」が主観的集合を問題にする（客観的集合ではないことを含意する）のに対し，「厳密に」は客観的集合を問題にする（主観的集合の設定を許さない）ためであると考えます。

4．「現実世界の事態の数量」という表現について

 ご指摘の通り，「「現実世界の事態の数量」との（不）一致」という表現，特に「現実世界」という表現は不適切でした。ご指摘いただきありがとうございました。第2稿では内容を修正した都合上，この表現自体を削除いたしました。

5．体験談では嘘と思われない程度の誇張や脚色がなされやすいこととの関わりについて

 「体験談」では「嘘と思われない程度の誇張や脚色がなされやすい」ため，「個人的な体験として語る場合、非該当例が許容されやすい」という見方については，筆者も関連性理論で言われる「ルース・トーク」（loose talk）に関する記述（Sperber and Wilson 1995 ^※^）を参考に検討いたしました。しかし，その場合，「ばかり」が遊離数量詞と共起する際に非該当例が許容されないことを説明できないため，この見方は採りませんでした。また，「ばかり」は「体験」の中から非該当例を排除した集合を設定すると筆者は捉えており，これは少なくとも一般的な「ルース・トーク」とは異なるものと考えております。

 ※    Sperber, D. & D. Wilson (1995) Relevance. Communication and Cognition.（内田聖二・中逵俊明・宋南先・田中圭子訳『関連性理論―伝達と認知―第2版』，1999年，研究者出版）

6．遊離数量詞によって非該当例を含んだ数が表示できない理由について

 筆者は，遊離数量詞が共起することで主観的な集合を形成する「事態」の数量が計量的に明示され，それによって集合の範囲が明確になると考えております。また，「事態」は「探索」と親和性があり，遊離数量詞が示す「事態」の数は「探索」の数と一致するのではないかと考えております。

 以上のご説明で十分なご回答になっているかは確信が持てませんが，現時点での筆者の考えを述べさせていただきました。

7．遊離数量詞による事態の数の明示は非該当例の存在が許容されなくなる根本的な原因ではないというご指摘について

 ご指摘いただきありがとうございます。確かに，第1稿では遊離数量詞による事態の数の明示が非該当例の存在が許容されなくなる根本的な原因であるかのように述べられております。しかし，これは筆者の述べ方の不備によるものであり，本来の意図はそうではありませんでした。

 筆者は，遊離数量詞による事態の数の明示は，非該当例の存在が許容されなくなることの根本的な原因ではなく，“「ばかり」は（主観的）集合の内部に非該当例が無いことを表す”ということを示唆する現象を観察することのできる操作（の1つ）であると考えております。第2稿では述べ方を全面的に修正し，この点が明確になるようにいたしました。

8．「数量詞」の範囲について

 ご指摘の通り，「朝からおかしな答案ばっかり、山ほど採点してこっちまでおかしくなる」の場合は非該当例（「まともな答案」）の存在が許容されやすい可能性があると思われます。また，先行研究の中には「数詞から成るもの」でない形式も「数量詞」の1つに数える論考があることも確認しております（宇都宮1995 ^※^など）。

 しかし，「数詞から成るもの」でない形式は一般的な数量詞（「数詞から成るもの」）と異なる性質を有しております。例えば，前者は後者に比べて位置的な制約が強いという点が挙げられます。

 ①    答案を 山ほど採点する。［NCQ］                  cf．答案を 30 枚採点する。

 ② ? 山ほどの答案を採点する。［QノNC］       cf． 30 枚の答案を採点する。

 ③ * 答案 山ほどを採点する。［NQC］                  cf．答案 30 枚を採点する。

 ご指摘いただいた前述の例は確かに興味深いものですが，今回はこのような相違点が認められることを考慮し，「数詞から成るもの」のみを「数量詞」と捉えることにいたしました。

 ※    宇都宮裕章（1995）「日本語数量詞体系の一考察」『日本語教育』87，pp.1-11．
